# Scaling of xylem and phloem transport capacity and resource usage with tree size

**DOI:** 10.3389/fpls.2013.00496

**Published:** 2013-12-05

**Authors:** Teemu Hölttä, Miika Kurppa, Eero Nikinmaa

**Affiliations:** Department of Forest Sciences, University of HelsinkiHelsinki, Finland

**Keywords:** carbon allocation, metabolic scaling, nitrogen allocation, phloem transport, pipe model, xylem transport

## Abstract

Xylem and phloem need to maintain steady transport rates of water and carbohydrates to match the exchange rates of these compounds at the leaves. A major proportion of the carbon and nitrogen assimilated by a tree is allocated to the construction and maintenance of the xylem and phloem long distance transport tissues. This proportion can be expected to increase with increasing tree size due to the growing transport distances between the assimilating tissues, i.e., leaves and fine roots, at the expense of their growth. We formulated whole tree level scaling relations to estimate how xylem and phloem volume, nitrogen content and hydraulic conductance scale with tree size, and how these properties are distributed along a tree height. Xylem and phloem thicknesses and nitrogen contents were measured within varying positions in four tree species from Southern Finland. Phloem volume, nitrogen amount and hydraulic conductance were found to be concentrated toward the branch and stem apices, in contrast to the xylem where these properties were more concentrated toward the tree base. All of the species under study demonstrated very similar trends. Total nitrogen amount allocated to xylem and phloem was predicted to be comparable to the nitrogen amount allocated to the leaves in small and medium size trees, and to increase significantly above the nitrogen content of the leaves in larger trees. Total volume, hydraulic conductance and nitrogen content of the xylem were predicted to increase faster than that of the phloem with increasing tree height in small trees (<~10 m in height). In larger trees, xylem sapwood turnover to heartwood, if present, would maintain phloem conductance at the same level with xylem conductance with further increases in tree height. Further simulations with a previously published xylem-phloem transport model demonstrated that the Münch pressure flow hypothesis could explain phloem transport with increasing tree height even for the tallest trees.

## Introduction

Structural-functional tree models describe formation and growth of new stem and branch axes as a function of their local environment and topological position within a tree (e.g., Sievänen et al., 2000). This approach has been shown to be a powerful tool in reproducing realistic tree architectures (Prusinkiewicz, 2004). Scaling rules, such as the pipe model (Shinozaki et al., 1964), have been used to quantify secondary growth to analyse tree mass balance during growth (e.g., Perttunen et al., 1996). While these empirical rules work well to describe average tree growth, they actually describe fundamental functional properties of trees related to material uptake and their long distance transport (e.g., West et al., 1999; McCulloh et al., 2003; Hölttä et al., 2011; Nikinmaa et al., 2013). Simultaneously, axial and secondary growth patterns determine the resource allocation within trees. Understanding the functional implications of axial scaling patterns is thus an essential feature of structural-functional tree models.

The long distance transport capacity of tree xylem has to be high enough to be able to deliver water from soil to leaves at the rate that it is transpired. Similarly, the long distance transport capacity of phloem has to match the production rate of carbon compounds assimilated in photosynthesis in the leaves. In addition, water and carbon exchange rates are strongly coupled as both occur through the same stomatal pores in leaves, although water use efficiency, i.e., the ratio of plant photosynthetic production to plant transpiration rate, may somewhat vary across tree species, climatic conditions and tree size (Cernusak et al., 2007). It is therefore reasonable to expect that the xylem and phloem transport capacities, i.e., conductance per leaf area, must also be coupled, but they may vary within species and tree sizes. It is well-recognized that xylem transport capacity will ultimately limit the photosynthetic production rate of a tree (Tyree and TSperry, 1988; Jones and Sutherland, 1991). Similarly, when the transport rate of photosynthates through the phloem is not able to keep up with the rate of photosynthesis, carbohydrates will start accumulating in the leaves and will cause down-regulation of photosynthesis and/or stomatal closure (e.g., Paul and Foyer, 2001; Nikinmaa et al., 2013).

The allometric relationships concerning the xylem have been under rigorous study for centuries [(Leonardo's notes MacCurdy, 2002; Huber, 1928; Zimmermann, 1983)]. Scaling relationships for xylem hydraulic conductance have been developed from theoretical basis and also tested empirically. Xylem sapwood cross-sectional area has been found to be conserved in branching, and also to be approximately linearly proportional to leaf area (Shinozaki et al., 1964; Berninger et al., 2005). Xylem conduit radius and conductivity has been found to increase from tree apex downwards, and also to increase with tree size (West et al., 1999; McCulloh et al., 2003) to compensate for the increased transport distance from soil to leaves. However, these changes in conductance compensate only partially the increased tree size (e.g., Mäkelä and Valentine, 2006).

Allometric relations of xylem have been of great interest not only from the water transport point of view, but also because the xylem construction and maintenance consumes a major proportion of the carbon budget of a tree (Mäkelä, 1986; Nikinmaa, 1992). The thick lignified xylem cell walls require large amounts of carbon over the long transport path in trees (Hacke et al., 2001). Xylem transport physiology and its connection with structure are relatively well-understood. The allometric relations concerning phloem transport have received much less attention. Anatomical and physiological measurements are much more difficult to conduct on the phloem, which is a more heterogeneous tissue and sensitive to external perturbations caused by direct measurements. Also, the transport rate of fluids, and therefore the required transport capacity, in the xylem typically exceeds that of the phloem by more than an order of magnitude (Hölttä et al., 2009). The few studies which have measured phloem dimensions report increased phloem allocation and decreased conduit dimensions, i.e., cross-sectional area (Quilhó et al., 2000), toward the tree apex. Phloem conduit size at tree base has also been found to increase with increases in tree height (Jensen et al., 2011, 2012; Mencuccini et al., 2011).

Although at static evaluation, the phloem comprises only a minor proportion of the carbon allocated to the tree, the yearly difference in allocation compared to xylem is not as large as functional phloem structures do not accumulate in the stem at the same extent as the xylem sapwood does. Further, phloem contains significant amounts of nitrogen since it is metabolically more active tissue with high protein content. Nitrogen is the most significant limitation of tree growth in boreal forests (Chapin et al., 1987; Bergh et al., 1999). Unlike total leaf area, which saturates to a rather stable value with increasing tree size at least at the stand level, e.g., Vanninen and Mäkelä (1999), the amount of xylem and phloem tissue should increase with tree height. Consequently, a growing proportion of tree's nitrogen could be expected to be found in the trunk and branches of a tree with increasing tree size (Helmisaari, 1995).

It has been suggested that, as a growth limiting substance, nitrogen is allocated optimally among leaves in tree crowns (Mooney and Gulmon, 1979; Field, 1983). The attention has focused especially on optimizing photosynthetic N-use efficiency (PNUE), the ratio of photosynthesis to leaf nitrogen content. In the optimal solution PNUE should be maximal and the same for all leaves independently of their position in the canopy (Mooney and Gulmon, 1979; Field, 1983). Yet, observed nitrogen allocation to leaves typically deviates from theoretical expectations (Field, 1983; Hirose and Werger, 1987; Evans, 1993; Hollinger, 1996; Kull, 2002; Wright et al., 2004). One overlooked aspect that may contribute to this deviation is that while foliar photosynthetic capacity depends on foliar nitrogen content, balanced structural design requires that there has to be sufficient capacity to transport photosynthates from the leaves as well. One should therefore consider also the nitrogen required for transporting substances to and from the leaves (in addition to the nitrogen needed for leaf functions) to understand the optimal within tree allocation of nitrogen.

We set out to determine allometric relations for the amounts of xylem and phloem, and their hydraulic conductances and nitrogen concentrations as a function of tree stem/branch diameters. We studied whether these relations were similar in four different species co-occurring in Southern Finland; birch (*Betula pendula*), aspen (*Populus tremula*), pine (*Pinus sylvestris*), and spruce (*Picea abies*). In general, the same biophysical constraints with respect to both xylem and phloem transport (in terms of mass flow driven by pressure gradient in a medium where the transport resistance is characterized by the transport distance and the cross-section area of the conducting tissues and the radius of the conduits within these tissues) have to apply to all of these species which are living in the same environmental conditions. We were interested whether the species specific differences, from e.g., differences in water use efficiency, phenological development, nitrogen requirements, and growth rates, would be large enough to reflect on these allometric relations despite the common driving gradients. Based on the allometric relations derived from the measurements, we derived scaling relations to estimate how whole tree xylem sapwood and phloem volume, nitrogen content and hydraulic conductance change as a function of tree size and how these properties are axially distributed within a tree. We also set out to estimate how much nitrogen was allocated to the xylem and phloem in comparison to the leaves. Then, using a previously published xylem and phloem transport model, we set out to answer whether the Münch pressure flow hypothesis would be a consistent explanation of phloem transport of photosynthates with increasing tree size.

## Materials and methods

### Derivation of scaling relationships as a function of stem/branch diameter

We expressed stem/branch diameter (*d*) as a power law function of distance from apex (*x*) Equation (1a). Xylem and phloem cross-sectional areas (*A*_*x*_ and *A*_*p*_), nitrogen concentrations (ρ_*N, x*_ and ρ_*N, p*_), conduit sizes (*r*_*x*_, *r*_*p*_), and the fraction of xylem and phloem cross-sectional areas occupied by conduits (ρ_*c, x*_ and ρ_*c, p*_) were expressed as power law functions of stem/branch diameter Equations (1b–i). The power function form was used since it is generally used to describe allometric variation in biological properties (e.g., Brown et al., 2004). See Table 1 for the list of symbols used in the manuscript.

**Table 1:** **Symbols used in the manuscript**.

*A*_*b*_	Cross-sectional area of bark
*A*_leaf_	Leaf area
*A*_*p*_	Phloem (living bark) cross-sectional area
*A*_*p*, tot_	Phloem cross-sectional area summed across all branches of a given diameter
*A*_*x*_	Xylem cross-sectional area
*A*_*x*, tot_	Xylem cross-sectional area summed across all branches of a given diameter
*A*_*sw*_	Xylem sapwood cross-sectional area
*A*_*hw*_	Xylem heartwood cross-sectional area
*d*	Branch/stem diameter
*K*_tot, *p*_	Total phloem conductance over the whole path-length (over whole tree)
*K*_tot, *x*_	Total xylem conductance over the whole path-length (over whole tree)
*k*_*p*_	Phloem hydraulic conductivity summed across all branches of a given diameter
*k*_*x*_	Xylem hydraulic conductivity summed across all branches of a given diameter
*L*	Tree height
*L*_0_	Distance from apex at which integration of whole tree properties are started from
*N*_*p*, tot_	Total amount of nitrogen in the phloem of a tree
*N*_*x*, tot_	Total amount of nitrogen in the xylem of a tree
*n*	Number of branches, i.e., furcations at a given height or stem/branch diameter
*R*_tot, *p*_	Total phloem resistance over the whole path-length (over whole tree)
*R*_tot, *x*_	Total xylem resistance over the whole path-length (over whole tree)
*r*_*p*_	Phloem conduit radius
*r*_*sw*, max_	Maximum sapwood radius (in cases where heartwood was taken into account)
*r*_*x*_	Xylem conduit radius
*V*_*p*, tot_	Total phloem volume in a tree
*V*_*x*, tot_	Total xylem volume in a tree
*x*	Distance from leaf apex
ρ_*c, p*_	Fraction of phloem occupied by the conducting sieve tubes
ρ_*c, x*_ I	Fraction of xylem occupied by the conducting sieve tubes
ρ_*N*, phloem_	Nitrogen concentration of phloem
ρ_*N*, bark_	Nitrogen concentration of outer bark
ρ_*N*, xylem_	Nitrogen concentration of xylem
γ, α_*i*_	Bases for scaling relations
δ, β_*i*_	Exponents for scaling relations

(1a) $$d=\gamma x^{\delta }.$$

(1b) $$A_{x}=\alpha_{1}*d_{1}^{\beta }=\alpha_{1}*{\left(\gamma x^{\delta }\right)}_{1}^{\beta }=\alpha_{1}\gamma_{1}^{\beta }x_{1}^{\delta \beta }$$

(1c) $$A_{p}=\alpha_{2}*d_{2}^{\beta }=\alpha_{2}*{\left(\gamma x^{\delta }\right)}_{2}^{\beta }=\alpha_{2}\gamma_{2}^{\beta }x_{2}^{\delta \beta }$$

(1d) $$\rho_{N,\;x}=\alpha_{3}*d_{3}^{\beta }=\alpha_{3}*{\left(\gamma x^{\delta }\right)}_{3}^{\beta }=\alpha_{3}\gamma_{3}^{\beta }x_{3}^{\delta \beta }$$

(1e) $$\rho_{N,\;p}=\alpha_{4}*d_{4}^{\beta }=\alpha_{4}*{\left(\gamma x^{\delta }\right)}_{4}^{\beta }=\alpha_{4}\gamma_{4}^{\beta }x_{4}^{\delta \beta }$$

(1f) $$r_{x}=\alpha_{5}*d_{5}^{\beta }=\alpha_{5}*{\left(\gamma x^{\delta }\right)}_{5}^{\beta }=\alpha_{5}\gamma_{5}^{\beta }x_{5}^{\delta \beta }$$

(1g) $$r_{p}=\alpha_{6}*d_{6}^{\beta }=\alpha_{6}*{\left(\gamma x^{\delta }\right)}_{6}^{\beta }=\alpha_{6}\gamma_{5}^{\beta }x_{6}^{\delta \beta }$$

(1h) $$\rho_{c,\;x}=\alpha_{7}*d_{7}^{\beta }=\alpha_{7}*{\left(\gamma x^{\delta }\right)}_{7}^{\beta }=\alpha_{7}\gamma_{7}^{\beta }x_{7}^{\delta \beta }$$

(1i) $$\rho_{c,p}=\alpha_{8}*d_{8}^{\beta }=\alpha_{8}*{\left(\gamma x^{\delta }\right)}_{8}^{\beta }=\alpha_{8}\gamma_{8}^{\beta }x_{8}^{\delta \beta }$$

We also took into account that xylem hydraulic conductance decreases radially inwards to the xylem tissue due to heartwood formation and/or decrease in the conduit size and connectivity to transpiring foliage (e.g., Melcher et al., 2003). For this, we assumed two extreme scenarios where (A) all of the xylem was conducting sapwood and (B) where conducting sapwood was restricted only to the outermost 2 cm of the xylem (*r*_*sw*, max_ = 2 cm) (e.g., Sellin, 1994). We reason that the actual amount of conducting sapwood, and thus xylem hydraulic conductance, must be in between these two extreme scenarios, and exploring the space between these two extremes describes how sensitive the scaling predictions are to changes in the radial profile of xylem hydraulic conductance. In addition, we assumed two extreme scenarios for xylem heartwood nitrogen content, where (A) heartwood nitrogen concentration was the same as in sapwood and (B) where heartwood nitrogen content was zero. Again, the actual amount of nitrogen in the xylem must lie in between these two extreme scenarios. A literature review by Meerts (2002) done on 71 angiosperm and 22 gymnosperm species reported that the nitrogen concentration of the heartwood was on average 76% of the nitrogen content of the sapwood, but varied a lot between species and studies.

We made an assumption that the cross-sectional area of the xylem sapwood is conserved at branching junctions. This assumption is from the pipe model theory formulated originally by Shinozaki et al. (1964). The pipe model assumption has been shown to hold reasonably well for the tree species used in our measurements (Kaufmann and Troendle, 1981; Ilomaki et al., 2003; Kantola and Mäkelä, 2004; Berninger et al., 2005), and also to result from maximizing the carbon use efficiency of xylem structure (Hölttä et al., 2011). Note that the original pipe model assumption, presented e.g., in Shinozaki et al. (1964), does not necessarily imply that xylem conduit radius is constant within a tree. We further assumed leaf area to be proportional to xylem cross-sectional sapwood area. This assumption is also from the Pipe model theory (Shinozaki et al., 1964) and is supported by experimental evidence (Berninger et al., 2005). Note that scaling of phloem and leaf properties are affected by the assumptions made about sapwood turnover to heartwood. This behavior stems from the pipe model assumption. The amount of heartwood affects the number of furcations [i.e., *n* in Equation (A18)], which in turn affects phloem properties [see Equations (A20), (A22), and (A24)]. The more heartwood there is, the higher the furcation number (*n*) is at any given height, and the more phloem tissue there is. The coefficients α_*i*_, β_*i*_, γ, and δ in Equation (1) were derived from measurements or from literature estimates. We used the allometric relations in Equation (1) to scale whole tree xylem and phloem volume, conductance and nitrogen amount with tree height for two of the measured species (one gymnosperm and one angiosperm); pine and aspen.

### Tree measurements

We harvested six birch (*Betula pendula*), aspen (*Populus tremula*), pine (*Pinus sylvestris*), and spruce (*Picea abies*) trees from forests surrounding Hyytiälä Forestry Field Station (61° 51' N, 24° 17' E, 180 m a.s.l.) and two from Ruotsinkylä research forests (60° 22' N, 25° 00' E) so that there were two trees of each species. Trees varied between 6.7 and 24.9 m in height and between 4.6 and 30.6 cm in breast height diameter and they grew in even-aged stands whose density varied between 8.6 and 28.3 m^2^/ha in basal area. The trees were harvested between May and October 2010. In addition, two additional pine trees were harvested in September 2013 for some additional nitrogen content measurements. All harvested trees were healthy. The cardinal points were marked to the sample trees before felling. Stem diameters were measured from various relative locations within the stem (1.0; 2.5; 5.0; 7.5; 10.0; 15.0; 20.0; 30.0; 40.0; 50.0; 60.0; 70.0; 80.0; 85.0, and 90.0%) in East to West and North to South directions. Bark thickness measurement were made from tree trunk at breast height and at 6 m height. We numbered all living branches from the base of the crown to the top of the tree and measured their heights from the tree base and marked their compass direction, length, and base diameters. Branch diameters were measured beyond noticeable basal swelling and the distance from tree trunk and apex were measured. The crown was divided into segments of height and compass directions and a total of 10–15 sample branches were selected from each tree from different heights and sides of the tree so that the branch size distribution was evenly represented in the sample. Only healthy appearing and non-damaged branches entered the sample.

We measured the length of sample branches from the cut surface to branch apex and measured over and under bark diameters from the base of the branch. Subsequently we divided each branch into segments. The first segment was from the base to the first fork and the following segments were between subsequent forking points. The length and one to three diameters were measured from each segment at 10, 50, and 90% of length along with the bark-less diameters. Thickness of the bark was calculated as the difference between under bark and over bark diameter divided by two. Altogether, we measured 4379 branch or stem diameters with and without bark between 0.9 and 276.2 mm with average and median diameter of 6.5 and 4.2 mm, respectively.

For measurements of the dimensions of the living bark we cut 85 stem disks from different heights of each tree (2.5–276.2 mm in diameter). The cut surfaces were sanded and scanned, and the thicknesses of the periderm and living bark were measured using a self-made image analyzer program. We used 62 samples from stem and branch disks (3.6–276 mm in diameter) for nitrogen the content measurements. The bark was removed from xylem. The periderm of bark was then removed, and the rest of the bark was termed as living bark, which consisted of the primary and secondary phloem, and vascular cambium. We dried the periderm and living bark samples at 60°C for 72 h and xylem samples for 120 h and ground all dried parts with an oscillating mill (Retsch MM400). The total nitrogen concentration in samples was determined by an element analyzer (Vario MAX CN) at the Department of Forest Sciences at the University of Helsinki.

Allometric equations were fitted to the measured data using non-linear regression between sample diameter and xylem and bark properties with Sigmaplot (Sigmaplot for Windows version 11.0). We used ANCOVA to compare similarities/differences amongst the different species in the above regressions with lm function of R version 2.13.0 after the data had been ln-transformed to correspond with the assumption of the test and verified the correspondence with the assumptions from residual plots.

### Estimation of the non-measured scaling relations from the literature

Xylem conduit radius (*r*_*x*_) was estimated to scale as *r*_*x*_ α*x*^0.25^ (e.g., West et al., 1999; Anfodillo et al., 2006). There are different estimates in the literature for the scaling of phloem conduit radius with tree height. We used a scaling relation of *r*_*p*_ α*x*^0.25^, i.e., the same as for the xylem. This is an intermediate scaling between the scaling exponent of 0.15 reported by Mencuccini et al. (2011), and 1/3 reported by Jensen et al. (2011). As distance to leaf apex is scaled from stem diameter, xylem and phloem conduit radius and conductance thus scale with stem/branch diameter, as has been found to be the case for xylem conduits (e.g., Zimmermann, 1983; Olson and Rosell, 2013). We assumed that a constant fraction of the xylem and phloem cross-sectional area was conducting lumen volume (the rest being conduit walls, parenchyma, etc.), i.e., β_7_ and β_8_ (exponents for the scaling equations for ρ_*c, x*_ and ρ_*c, p*_) were equal to zero. This is in agreement with a formulation described e.g., in Savage et al. (2010).

### Sensitivity analysis for the scaling relations

To demonstrate the sensitivity of the scaling of xylem and phloem volume, nitrogen content, and hydraulic conductance to the values of the measured/estimated scaling exponents we conducted simulations where the scaling exponents β_1_, β_2_, β_3_, β_4_, β_5_, β_6_, and δ were varied simultaneously in random (from a linear distribution) between 75 and 125% of the values in comparison to values presented in Table 2. Simultaneously, the maximum sapwood depth (*r*_*sw*, max_) was varied between 2 and 200 cm.

**Table 2:** Measured properties as a function of stem/branch diameter for each species from the measurements: *P* = *B* * *d*^*E*^, where *P* is the property under consideration, *B* is the base, and *E* is the exponent.

**Quantity**	**Corresponding scaling coefficient (intercept and slope)**	**Species**	**Intercept (units)**	**Slope**	***R*^2^**	***P***	***n***
Bark cross-sectional area	–	Birch	0.45 mm^2^	1.60	0.98	***	553
		Pine	0.38 mm^2^	1.61	0.88	***	2050
		Spruce	0.52 mm^2^	1.59	0.97	***	1371
		Aspen	0.61 mm^2^	1.53	0.97	***	399
		All	0.31 mm^2^	1.69	0.90	***	4377
Xylem cross-sectional area	α_1_ and β_1_	Birch	0.67 mm^2^	2.01	0.999	***	555
		Pine	0.82 mm^2^	1.98	0.999	***	2052
		Spruce	0.55 mm^2^	2.05	0.999	***	1372
		Aspen	0.71 mm^2^	2.00	0.999	***	401
		All	0.69 mm^2^	2.00	0.999	***	4379
Phloem cross-sectional area	α_2_ and β_2_	Birch	0.32 mm^2^	1.59	0.99	***	10
		Pine	0.75 mm^2^	1.27	0.98	***	62
		Spruce	0.0086 mm^2^	2.35	0.93	***	10
		Aspen	0.99 mm^2^	1.3	0.94	***	37
		All	1.97 mm^2^	1.11	0.94	***	130
Xylem nitrogen content	α_3_ and β_3_	Birch	0.11%	0.21	0.099	NS	4
		Pine	2.02%	−0.61	0.86	***	27
		Spruce	0.15%	−0.021	0.0033	NS	8
		Aspen	0.68%	−0.34	0.80	**	9
		All	1.92%	−0.59	0.85	***	48
Phloem nitrogen content	α_4_ and β_4_	Birch	7.1%	−0.59	0.86	***	12
		Pine	1.4%	−0.22	0.73	***	28
		Spruce	0.87%	−0.10	0.0387	NS	9
		Aspen	22 %	−0.79	0.90	***	13
		All	1.48%	−0.20	0.38	***	62
Distance from apex	γ and δ	Birch	115 mm	1.00	0.97	***	370
		Pine	61 mm	1.029	0.97	***	1168
		Spruce	85 mm	0.98	0.96	***	1144
		Aspen	73 mm	1.05	0.97	***	483
		All	87 mm	0.98	0.94	***	3165

Stem/branch diameter (d) is in units of millimeters (mm). Significance levels

*** P < 0.001,

** P < 0.01, NS = P > 0.05.

### Simulations of xylem and phloem transport with a numerical model

Xylem and phloem transport and the resulting within tree pressure gradients were simulated using the scaling relations obtained and a previously published xylem and phloem transport model (Hölttä et al., 2009). The model calculates xylem and phloem pressure and sugar concentrations and their within tree axial gradients in steady state. Pressure differences drive xylem and phloem transport, i.e., flow is proportional to pressure gradient, and water potential equilibrium is maintained between the xylem and phloem. Phloem sap viscosity was made to be sugar concentration dependent. Transpiration rate, phloem loading (made equal to photosynthesis rate) and unloading rates and soil water potential were given as boundary conditions, and xylem and phloem hydraulic conductance and tree height were given as structural parameters. The position of phloem unloading could be varied in the transport model so that we ran simulations where phloem unloading was made to occur either evenly along the phloem transport pathway or exclusively in the roots. We did three simulations with the model. (1) We used the model to calculate the axial xylem and phloem pressure and sugar concentration gradients taking into account the axial distribution of xylem and phloem tissue and their specific conductivity. We used a 10 m pine with a maximum sapwood depth of 2 cm as an example, and took the axial distribution of xylem and phloem conductivity from the equations shown in the Appendix A3 and demonstrated in Figure 7. (2) We modeled how phloem tissue should be distributed axially in order to minimize turgor pressure difference between the leaves and roots, i.e., source and sink, for a fixed phloem volume at the whole tree level. In other words, the total amount of phloem tissue was preserved, but was distributed unevenly as a function of axial position. (3) We simulated how phloem turgor pressure difference between the leaves and roots would change as a function of increasing tree height, and whether phloem transport would be able to function according to the Münch pressure flow hypothesis when trees become taller. For this, we used the scaling relations for whole tree xylem and phloem hydraulic conductance as a function of tree height derived in Appendixes A1 and A2 (and demonstrated in Figure 4 and Table 4) for the case of pine. In this simulation, leaf gas exchange rates (i.e., transpiration and photosynthesis rates) were determined for each tree height so that leaf xylem water potential always remained at a constant value, i.e., we assumed isohydric behavior. Leaf water potential was held at −2.0 MPa, which is a typical value for Scots Pine in Hyytiälä Forestry Field Station in summer conditions (Martinez-Vilalta et al., 2009). Photosynthesis rate was made to be proportional to transpiration rate. Water use efficiency was set to 250, a typical value for Scots Pine in Hyytiälä Forestry Field Station (e.g., Hari and Mäkelä, 2003). The absolute value for phloem conductance was chosen so that the turgor pressure difference between the leaves and roots obtained a reasonable value, ~0.7 MPa for the case of unloading in the soil for a 10 m tree. Note that this resulted in different initial values for phloem conductance between the cases where no heartwood was assumed and the case where the maximum sapwood depth was set at 2 cm. We also varied the initial value of phloem conductive and distribution of phloem unloading to see their effects on the results.

## Results

### Scaling of whole tree properties from allometric equations

The equations obtained for the scaling of xylem and phloem properties as a function of tree height (*L*) (starting from a distance *L*_0_ from leaf apex) are as follows

(2a) $$n(x)={\left(\frac{L}{x}\right)}^{\delta \beta_{1}}$$

(2b) $$V_{x,\text{ tot}}=\alpha_{1}\gamma^{\beta_{1}}\left(L^{\delta \beta_{1}+1}-L_{0}^{\delta \beta_{1}+1}\right)$$

(2c) $$V_{p,\text{ tot}}=\frac{L^{\delta \beta_{1}}\alpha_{2}\gamma^{\beta_{2}}}{\delta \beta_{2}-\delta \beta_{1}+1}\left(L^{\delta \beta_{2}-\delta \beta_{1}+1}-L_{0}^{\delta \beta_{2}-\delta \beta_{1}+1}\right)$$

(2d) $$N_{x,\text{ tot}}=\frac{\alpha_{1}\gamma^{\beta_{1}}\alpha_{3}\gamma^{\beta_{3}}\rho_{x}}{\delta \beta_{3}+1}\left(L^{\delta \beta_{1}+\delta \beta_{3}+1}-L_{0}^{\delta \beta_{1}+\delta \beta_{3}+1}\right)$$

(2e) $$N_{p,\text{ tot}}=\frac{L^{\delta \beta_{1}}\alpha_{2}\gamma^{\beta_{2}}\alpha_{4}\rho_{N}\gamma^{\beta_{4}}}{\delta \beta_{4}+\delta \beta_{2}-\delta \beta_{1}+1}\left(L^{\delta \beta_{4}+\delta \beta_{2}-\delta \beta_{1}+1}-L_{0}^{\delta \beta_{4}+\delta \beta_{2}-\delta \beta_{1}+1}\right)$$

(2f) $$N_{\text{leaf},\text{ tot}}=\frac{\alpha_{1}\gamma^{\beta_{1}}\left(L^{\delta \beta_{1}+1}-L_{0}^{\delta \beta_{1}+1}\right)}{L}\;C_{ls}\;\rho_{\text{SLA}}\;\rho_{N,\text{ leaf}}$$

(2g) $$\begin{array}{l} K_{x,\text{ tot}}^{-1}={\left(L^{\delta \beta_{1}}\alpha_{1}\alpha_{7}\alpha_{5}^{2}\gamma^{\beta_{1}+\beta_{7}+2\beta_{5}}\right)}^{-1}\frac{1}{-\delta \beta_{7}-2\delta \beta_{5}+1}\\ \;\left(L^{-\delta \beta_{7}-2\delta \beta_{5}+1}-L_{0}^{-\delta \beta_{7}-2\delta \beta_{5}+1}\right) \end{array}$$

(2h) $$\begin{array}{l} K_{p,\text{ tot}}^{-1}={\left(L^{\delta \beta_{1}}\alpha_{2}\alpha_{8}\alpha_{6}^{2}\gamma^{\beta_{2}+\beta_{8}+2\beta_{6}}\right)}^{-1}\frac{1}{-\delta \beta_{2}+\delta \beta_{1}-\delta \beta_{8}-2\delta \beta_{6}+1}\\ \;\left(L^{-\delta \beta_{2}+\delta \beta_{1}-\delta \beta_{8}-2\delta \beta_{6}+1}-L_{0}^{-\delta \beta_{2}+\delta \beta_{1}-\delta \beta_{8}-2\delta \beta_{6}+1}\right) \end{array}$$

where *n* is the number of branches, i.e., furcations, at a distance *x* from the leaf apex, *V*_*x*, tot_ is the total xylem volume in a tree, *V*_*x*, tot_ is the total xylem volume in a tree, *N*_*x*, tot_ is the total amount of nitrogen in the xylem in a tree, *N*_*p*, tot_ is the total amount of nitrogen in the phloem in a tree, *N*_leaf, tot_ is the total amount of nitrogen in the leaves of a tree, *K*_*x*, tot_ is the whole tree hydraulic conductance of the xylem, *K*_*x*, tot_ is the whole tree hydraulic conductance of the phloem. The derivation of the equations is presented in the Appendix A1. These equations apply only to the case without heartwood. The numerical equations for the whole tree scaling relations including sapwood to heartwood turnover are shown in Appendix A2. *L*_0_ was set to 0.1 m, except in the case of aspen phloem nitrogen content, in which *L*_0_ was set to 1.0 m. The values of 0.1 m and 1 m for *L*_0_ correspond to branch diameter of ~2 and 20 mm, respectively (see Figure 2C). Xylem and phloem properties were given constant values at branches than smaller than this. The values for *L*_0_ were chosen large enough so that we had measurements from branches of corresponding diameter.

### Tree measurements

#### Whole bark vs. diameter

**Figure 1:**
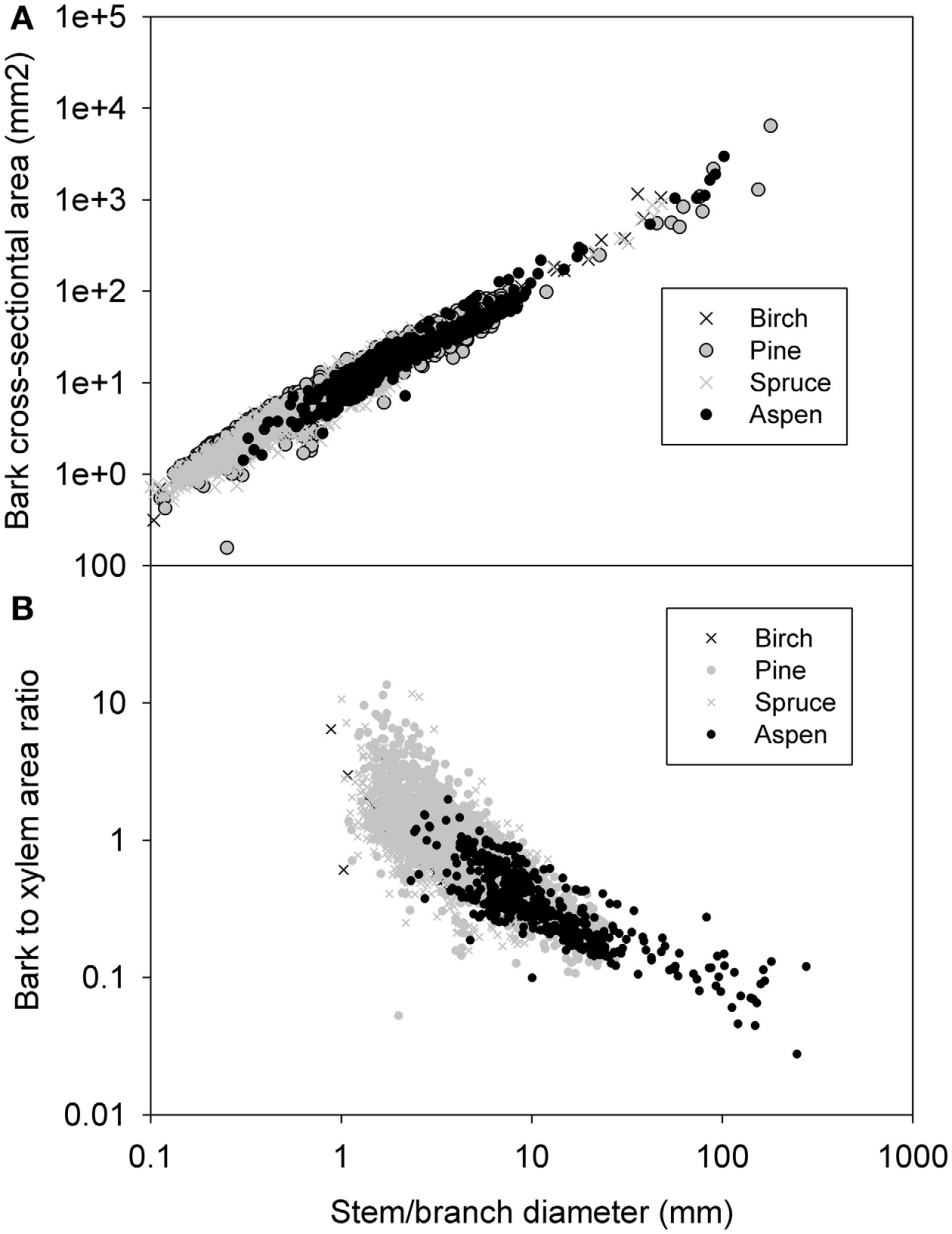
**Measured bark cross-sectional (A) and the ratio of bark to xylem cross-sectional area (B) as a function of stem/branch diameter**.

The cross-sectional area of the whole bark (*A*_*b*_), i.e., inner plus outer bark, increased with stem/branch diameter following a relationship *A*_*b*_ = 0.31 *d*^1.68^ when all the data was pooled together (Figure 1A). The scaling exponent ranged from 1.5344 in aspen to 1.59–1.61 in spruce, birch and pine with all relations being highly significant (see Table 2). The scaling exponents were rather close to each other across the species. When testing the difference, the logarithmic transformation changed the exponents somewhat (1.45, 1.50, 1.52, and 1.60 in pine, birch, aspen, and spruce, respectively). Out of these values, only the spruce exponent was very significantly different (*p* < 0.001) from the birch exponent. The ratio of whole bark to xylem diameter showed a consistent trend of decreasing with increases in stem/branch diameter, following a relationship *A*_*b*_/*A*_*x*_ = 3.32*d*^−0.94^ (Figure 1B). The relation of whole bark diameter against stem/branch diameter was not used in the scaling predictions since it does no. Instead, the bark was divided into outer and inner bark, and the latter represents the functional phloem tissue.

#### Inner bark and xylem cross-sectional area vs. diameter and stem taper

**Figure 2:**
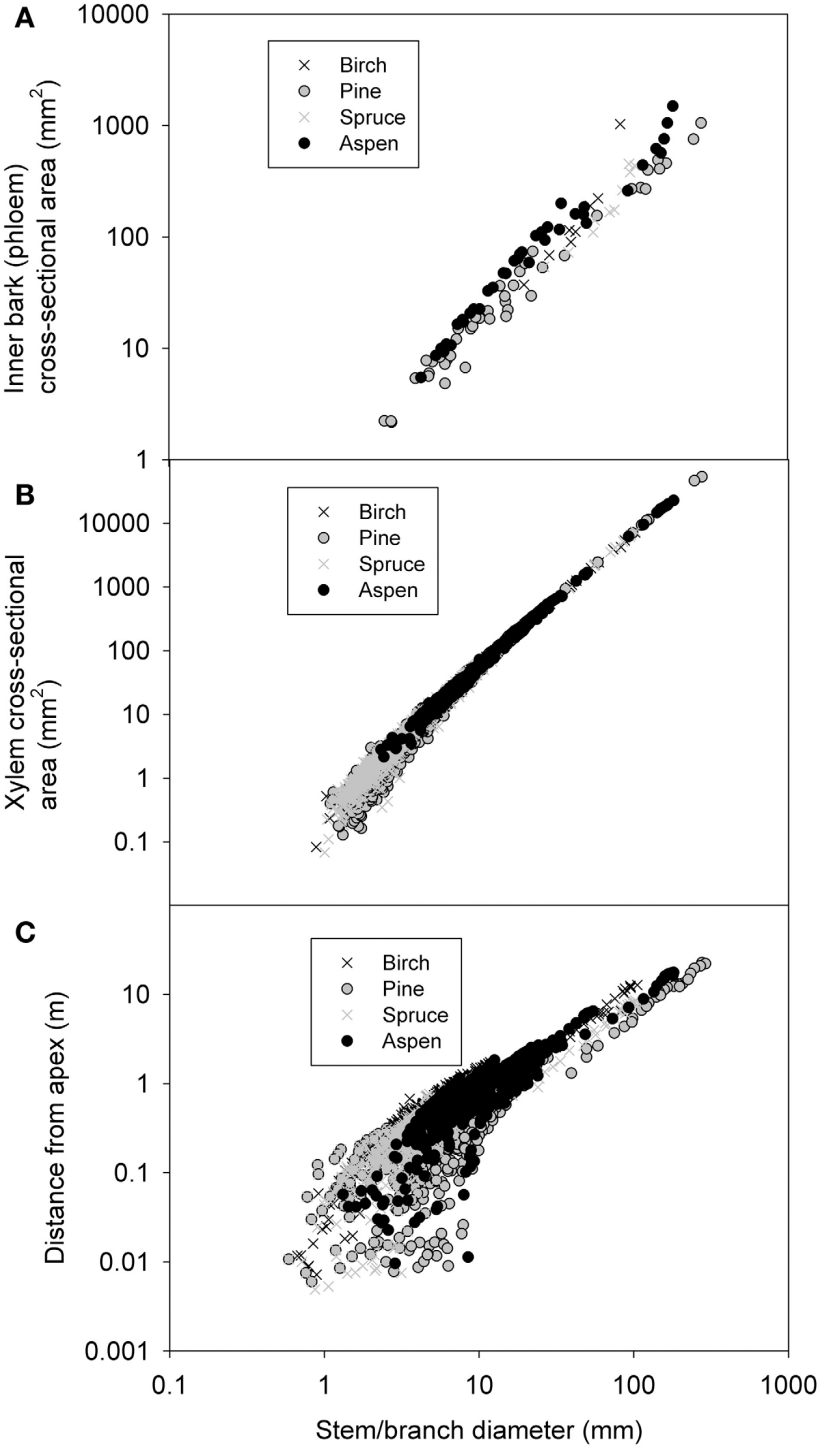
**Measured inner bark (i.e., phloem) cross-sectional area (A), xylem cross-sectional area (B), and distance from leaf apex (C) as a function of stem/branch diameter**.

For inter-species comparison of inner bark thickness there was sufficient data for aspen and pine. Their exponents were not significantly different from each other in the ln-transferred data. Pooling all species data together for the non-linear regression yielded *A*_*p*_ = 1.2*d*^1.286^ for the relation between bark and branch/stem diameter (Figure 2A, Table 2). The cross-sectional area of the xylem (*A*_*x*_) increased with stem/branch diameter in slightly different manner between the species (see Table 2). Pooling the data together yields *A*_*x*_ = 0.68*d*^2.009^ for the relation between xylem tissue and branch/stem diameter (Table 2, Figure 2B). When all data was pooled together, the cross-sectional areas of xylem and phloem were equal at branch diameters of ~2.5 mm. Phloem thickness resulted in saturating to a very constant value (phloem thickness = 0.88 ^*^d^0.082^, all species combined together, not shown). When data from the main stems plus the first order branches was pooled together, the distance from leaf apex was found to scale with stem/branch diameter following α *d*^0.98^ (Figure 2C).

#### Nitrogen content

**Figure 3:**
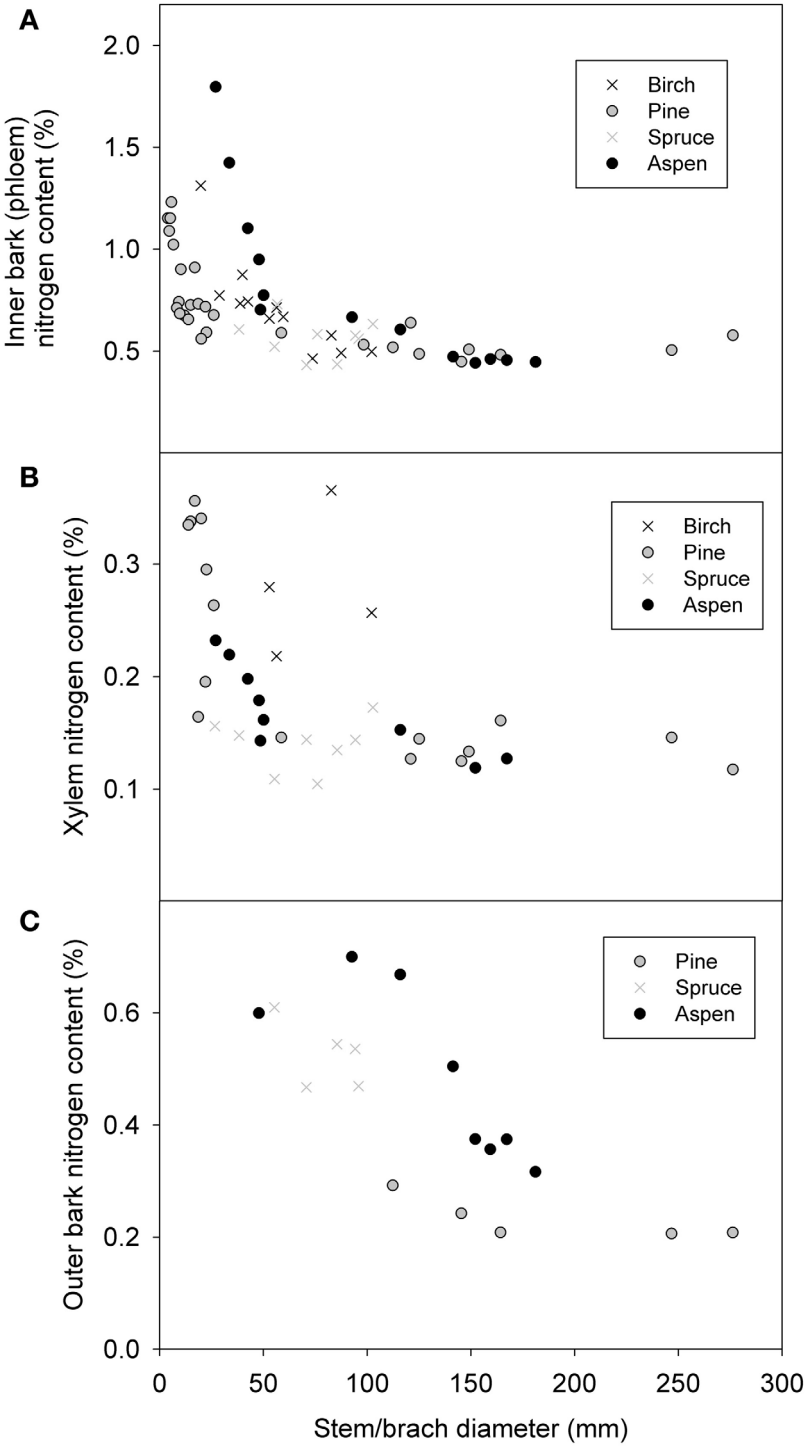
**Nitrogen concentration of living bark (A), xylem (B), and whole bark (C) as a function of stem/branch diameter.** The data measurement points in living bark and xylem were from 8 trees (4 species).

Nitrogen content increased clearly with decreasing stem diameter in both the living bark and the whole bark, but remained fairly constant for the xylem (Figure 3). When all species were pooled together relations of ρ_*N*, phloem_ = 0.0148 d^−0.20^ for the inner bark, and ρ_*N*, xylem_ = 0.0192 d^−0.59^ for xylem (Table 2). As there were only four birch values that differed clearly from the rest we also calculated the relationship without birch yielding ρ_*N*, xylem_ = 0.0021 d^−0.64^. While all species seemed to follow similar pattern for the living bark, there seemed to be a level difference for the whole bark so that there was the most nitrogen in the aspen bark and least in pine bark for the same diameter. The phloem nitrogen concentrations in the smallest branch diameters were found to be very close to the nitrogen concentration found in the foliage of the corresponding species in pine and aspen (compare Figure 2A to ρ_*N*, leaf_ in Table 3).

**Table 3:** **The scaling coefficients of xylem and phloem properties as a function of stem/branch diameter used in the scaling Equation (1)**.

**Scaling coefficient**	**Symbol**	**Value used for pine**	**Value used for aspen**	Measured/estimated from literature^*^
Base for stem/branch diameter as a function of distance from leaf apex (mm)	γ	15.8 mm	15.6 mm	Measured
Base for xylem cross-sectional area as a function of stem/branch diameter	α_1_	0.82	0.71	Measured
Base for phloem cross-sectional area as a function of stem/branch diameter	α_2_	0.75	0.99	Measured
Base for xylem nitrogen concentration as a function of stem/branch diameter	α_3_	2.02	0.68	Measured
Base for phloem nitrogen concentration as a function of stem/branch diameter	α_4_	1.4	22.1	Measured
Base for xylem conduit radius as a function of stem/branch diameter	α_5_	1 unit less	1 unit less	Irrelevant as only relative values are shown
Base for phloem conduit radius as a function of stem/branch diameter	α_6_	1 unit less	1 unit less	Irrelevant as only relative values are shown
Base for xylem conduit density (ratio of conduit cross-sectional area to tissue cross-section area) as a function of stem/branch diameter	α_7_	1 unit less	1 unit less	Irrelevant as only relative values are shown
Base for phloem conduit density (ratio of conduit cross-sectional area to tissue cross-section area) as a function of stem/branch diameter	α_8_	1 unit less	1 unit less	Irrelevant as only relative values are shown
Exponent for stem/branch diameter as a function of distance from leaf apex	δ	0.97	0.97	Measured
Exponent for xylem cross-sectional area as a function of stem/branch diameter	β_1_	1.98	2.00	Measured
Exponent for phloem cross-sectional area as a function of stem/branch diameter	β_2_	1.27	1.31	Measured
Exponent for xylem nitrogen concentration as a function of stem/branch diameter	β_3_	−0.61	−0.34	Measured
Exponent for phloem nitrogen concentration as a function of stem/branch diameter	β_4_	−0.22	−0.80	Measured
Exponent for xylem conduit radius as a function of stem/branch diameter	β_5_	0.25	0.25	West et al., 1999
Exponent for phloem conduit radius as a function of stem/branch diameter	β_6_	0.25	0.25	West et al., 1999; Jensen et al., 2011; Mencuccini et al., 2011
Exponent for xylem conduit density (ratio of conduit cross-sectional area to tissue cross-section area)as a function of stem/branch diameter	β_7_	0	0	Savage et al., 2010
Exponent for phloem conduit density (ratio of conduit cross-sectional area to tissue cross-section area) as a function of stem/branch diameter	β_8_	0	0	Assumed to be the same as for the xylem
Leaf to sapwood area ratio C_*ls*_	C_*ls*_	3400 m^2^/m^2^	2700 m^2^/m^2^	Hoffmann and Usoltsev, 2002; Martinez-Vilalta et al., 2009
Specific leaf area	ρ_SLA_	300 g/m^2^	80 g/m^2^	Niinemets, 1997; Martinez-Vilalta et al., 2009
Leaf nitrogen content	ρ_*N*, leaf_	1.2%	2.4%	Niinemets, 1997; Martinez-Vilalta et al., 2009

Stem/branch diameter (d) is in units of millimeters (mm).

#### Whole tree scaling relations

**Figure 4:**
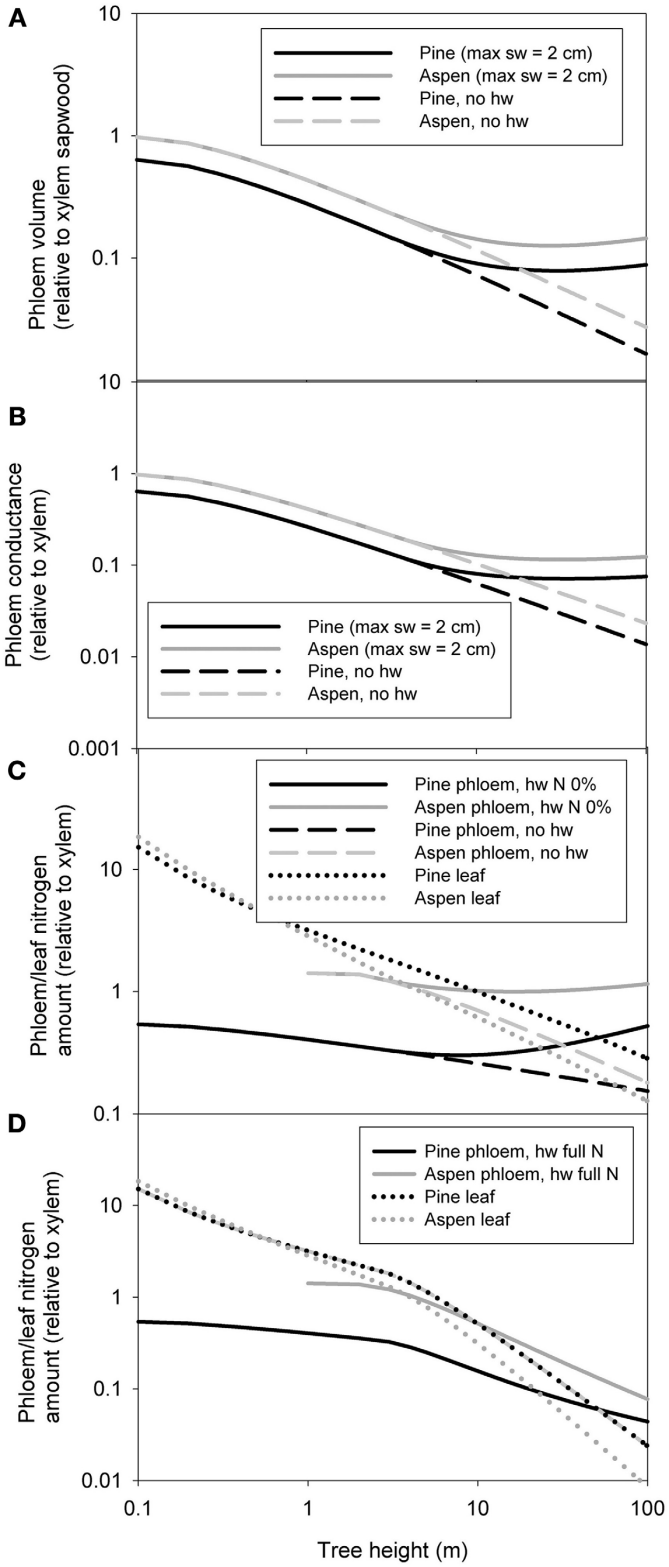
**The predictions for the whole tree phloem volume in relation to xylem sapwood volume (A), phloem hydraulic conductance in relation xylem hydraulic conductance (B), total phloem and leaf nitrogen content in relation to xylem hydraulic content for the scenarios in which the heartwood has the same nitrogen content as the sapwood and for the case of no heartwood (C), and phloem and leaf nitrogen content in relation to xylem hydraulic content for the case where the heartwood has the same nitrogen content as the sapwood (D).** “sw = 2 cm” refers to a scenario where maximum sapwood depth was assumed to be 2 cm and the heartwood did not contain any nitrogen, “no hw” to a scenario of no heartwood formation, i.e., all of the xylem was assumed to be conducting, and “hw full N” a scenario where maximum sapwood depth was assumed to be 2 cm and the heartwood nitrogen content was the same as that of the sapwood. In **(B)** the same area-specific conductivity was assumed for xylem and phloem.

**Figure 5:**
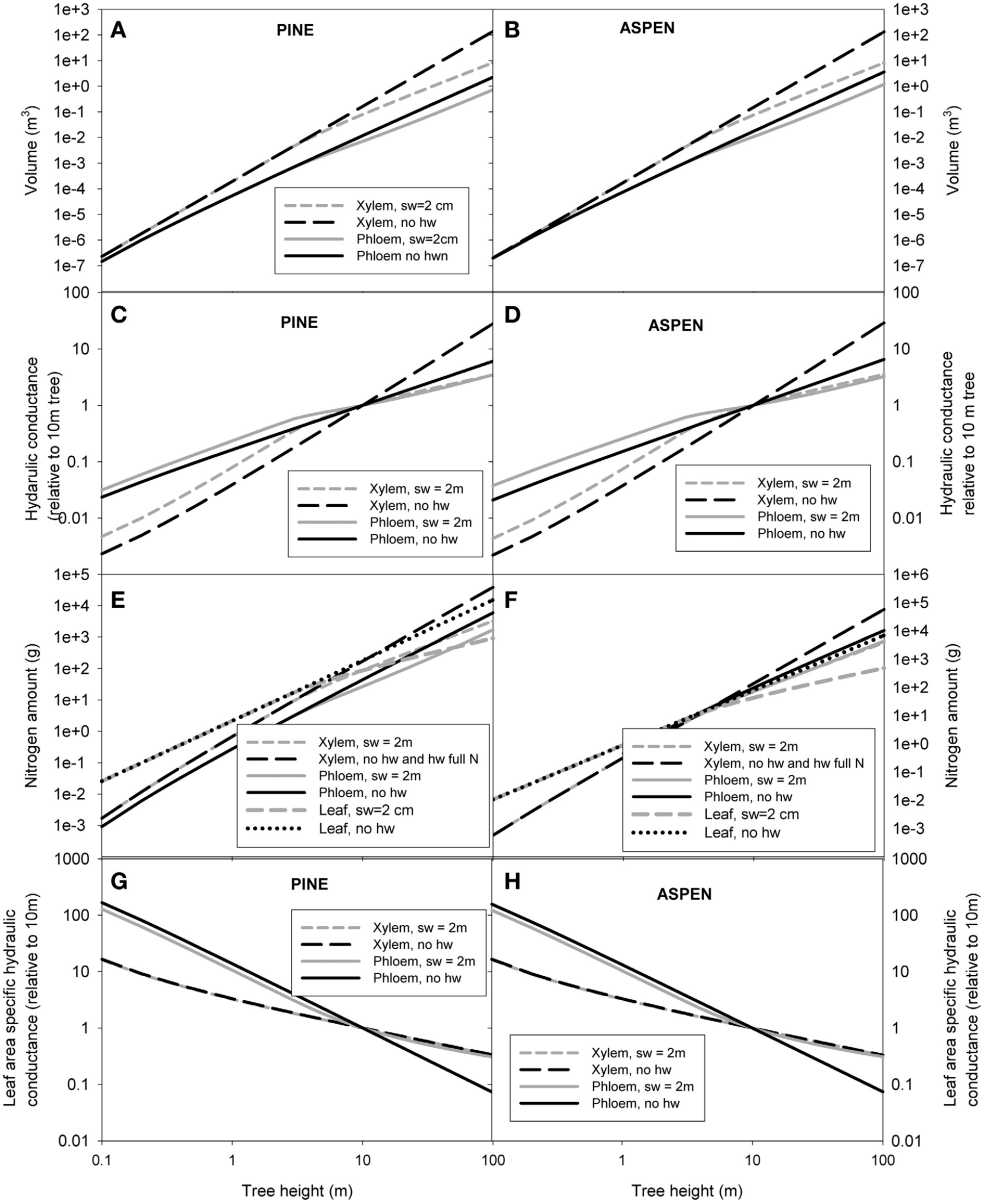
**The predictions for the absolute values for whole tree volume of xylem and phloem (A,B), hydraulic conductance of xylem and phloem (C,D), nitrogen content of xylem, phloem and leaves (E,F), and hydraulic conductance of xylem and phloem per leaf area (G,H) as a function of tree height.** “sw = 2 cm” refers to a scenario where maximum sapwood depth was assumed to be 2 cm and the heartwood did not contain any nitrogen, “no hw” to a scenario of no heartwood formation, i.e., all of the xylem was assumed to be conducting, and “hw full N” a scenario where maximum sapwood depth was assumed to be 2 cm and the heartwood nitrogen content was the same as that of the sapwood.

Whole tree scaling relation predictions were made for two example species: pine and aspen. The allometric relations used in the scaling of whole tree xylem and phloem volume, nitrogen content and hydraulic conductance are presented in Table 3. Some of the parameter values (α_1 − 4_, β_1 − 5_, γ, and δ) used in the scaling were taken from the measurements, while the rest (α_5_ − α_8_, β_6_ − β_9_, C_*ls*_, ρ_SLA_, and ρ_*N*, leaf_) were estimated from literature. Figure 4 shows the scaling relations for phloem and leaf properties in relation to the xylem properties, and Figure 5 shows the absolute values for xylem, phloem and leaf properties. Whole tree phloem volume and hydraulic conductance decreased more sharply in comparison to whole tree xylem sapwood volume and hydraulic conductance with increases in tree height in small tree heights (<10 m in height), and also in larger trees in the absence of xylem heartwood formation (Figures 4A,B, 5A–D,G,H). However, at trees larger than ~10 m in height, xylem heartwood formation according to the maximum 2 cm sapwood depth scenario was found to maintain phloem conductance and volume at approximately a constant proportion of the xylem sapwood with increases in tree height. Aspen had a larger amount of phloem and higher phloem to xylem ratio in relation to pine. Leaves were the largest sink of nitrogen in small trees, but xylem and phloem exceeded the leaves as a nitrogen sink with increases in tree height (Figures 4C,D, 5E,F). The total nitrogen content of the phloem was smaller than that of the xylem in pine and large aspen trees. The total nitrogen content of the phloem exceeded the xylem nitrogen content in small aspen trees (Figures 4C,D, 5E,F). Assumptions on heartwood proportions and nitrogen content of the heartwood caused the relative nitrogen contents between the tissues to vary strongly. When heartwood nitrogen concentration was low (“hw N 0%” in Figure 4C), phloem remained an important nitrogen sink relative to xylem even in large trees. However, when there was no heartwood, or the nitrogen content of heartwood was assumed to be same as that of the sapwood, then the role of the phloem as a nitrogen sink decreased in relation to xylem with increases in tree size. Table 4 present the absolute values for scaling of tree xylem and phloem volume, nitrogen content, conductance, and leaf area-specific conductance as a function of tree size. Note that scaling is not strictly allometric [see Equation (2) and Appendix A2], although very close to it, for each case.

**Table 4:** **The results for scaling of tree properties as a function of tree height (*L*)**.

**Property**	**Pine xylem**	**Pine phloem**	**Aspen xylem**	**Aspen phloem**
Volume (no hw)	2*10^−5^*L^2.93^	6.5*10^−5^*L^2.27^	2*10^−5^*L^2.94^	9.0*10^−5^*L^2.31^
Volume (sw = 2 cm)	9*10^−5^*L^1.99†^	4.4*10^−5^*L^2.11^	9*10^−5^*L^1.99†^	6.2*10^−5^*L^2.14^
Nitrogen content (no hw)	0.79*L^2.3467^	0.35* L^2.11^	0.34*L^2.61^	1.0488*L^1.9954^
Nitrogen content (sw = 2 cm, hw full N)	0.79*L^2.3467^	0.30*L^1.88^	0.34*L^2.61^	1.03*L^1.82^
Nitrogen content (sw = 2 cm, hw N 0%)	2.52*L^1.56^	0.30*L^1.88^	1.54*L^1.70^	1.03*L^1.82^
Conductance (sw = 2 cm)	α *L*^0.54^	α *L*^0.56^	α *L*^0.55^	α *L*^0.53^
Conductance (no hw)	α *L*^1.45^	α *L*^0.78^	α *L*^1.47^	α *L*^0.81^
Conductance/leaf area (no hw)	α *L*^−0.64^	α *L*^−1.07^	α *L*^−0.64^	α *L*^−1.06^
Conductance/leaf area (sw = 2 cm)	α *L*^−0.63^	α *L*^−1.06^	α *L*^−0.63^	α *L*^−1.05^
Leaf nitrogen content, no hw	2.14*L^1.93^		0.91*L^1.94^	
Leaf nitrogen content sw = 2 cm	9.40*L^1.00^		4.57*L^1.01^	

Only the exponents were estimated for the conductances. “(sw = 2 cm, hw N 0%)” refers to a scenario where maximum sapwood depth was assumed 2 cm and heartwood was assumed not to contain any nitrogen, “sw = 2 cm, hw full N” a scenario where maximum sapwood depth was assumed to be 2 cm and the heartwood nitrogen content was the same as that of the sapwood, and “no hw” refers to a scenario of no heartwood formation, i.e., all of the xylem was assumed to be conducting.

† Sapwood volume

#### Sensitivity analysis for the scaling relations

**Figure 6:**
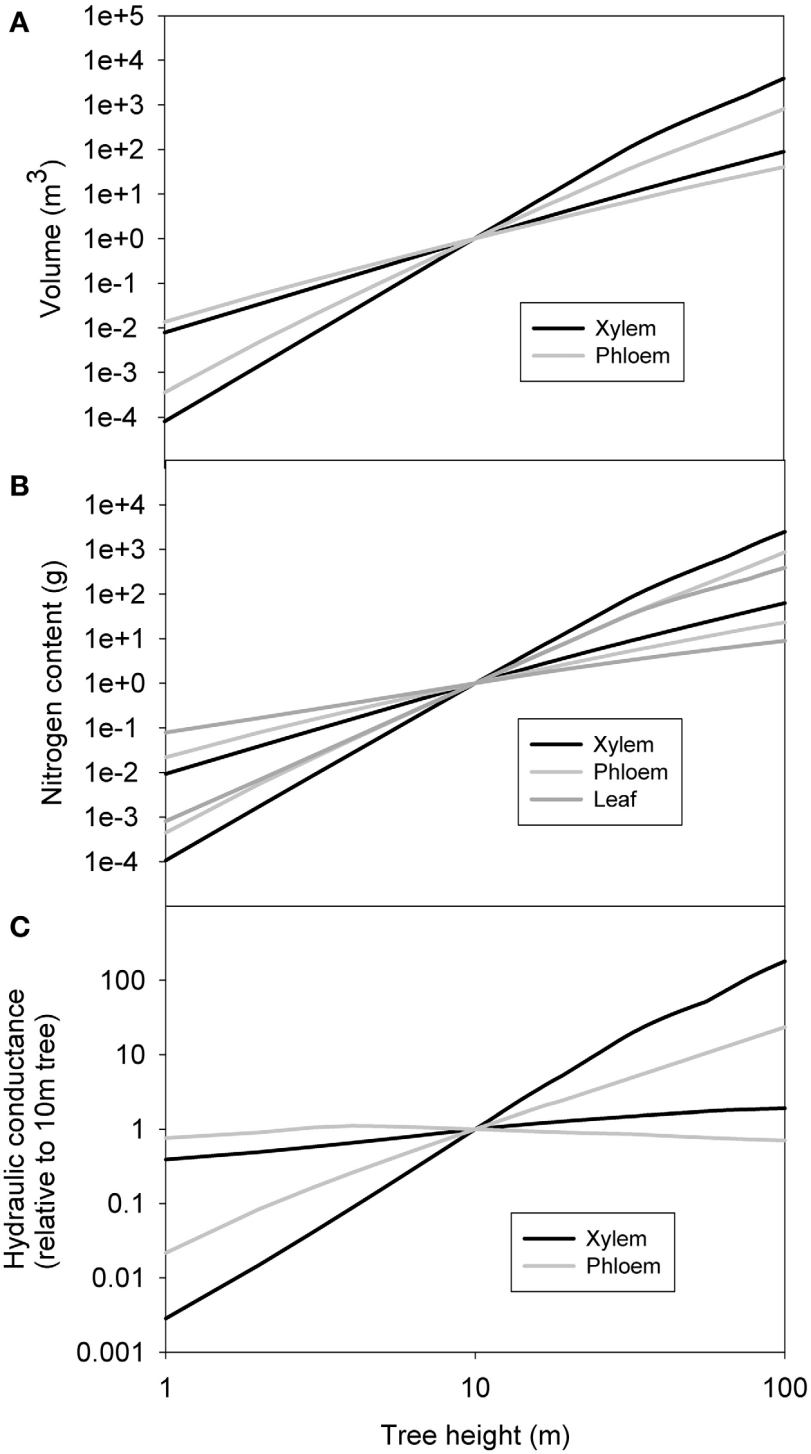
**The minimum and maximum xylem sapwood and phloem volume (A), nitrogen content (B) and conductance (C) in relation to a 10 m tree obtained from the sensitivity analysis done with 1000 parameter combinations.** Also total leaf nitrogen content is shown in **(B)**.

Figure 6 shows the minimum and maximum xylem and phloem volume, nitrogen content and conductance in relation to a 10 m tree obtained from the sensitivity analysis done with 1000 parameter combinations. The general trends within remained unchanged, although the xylem, phloem and leaf properties overlapped with each other. Xylem and leaf properties seemed to be more sensitive to parameter combination than those of phloem.

#### Axial distribution within a tree

**Figure 7:**
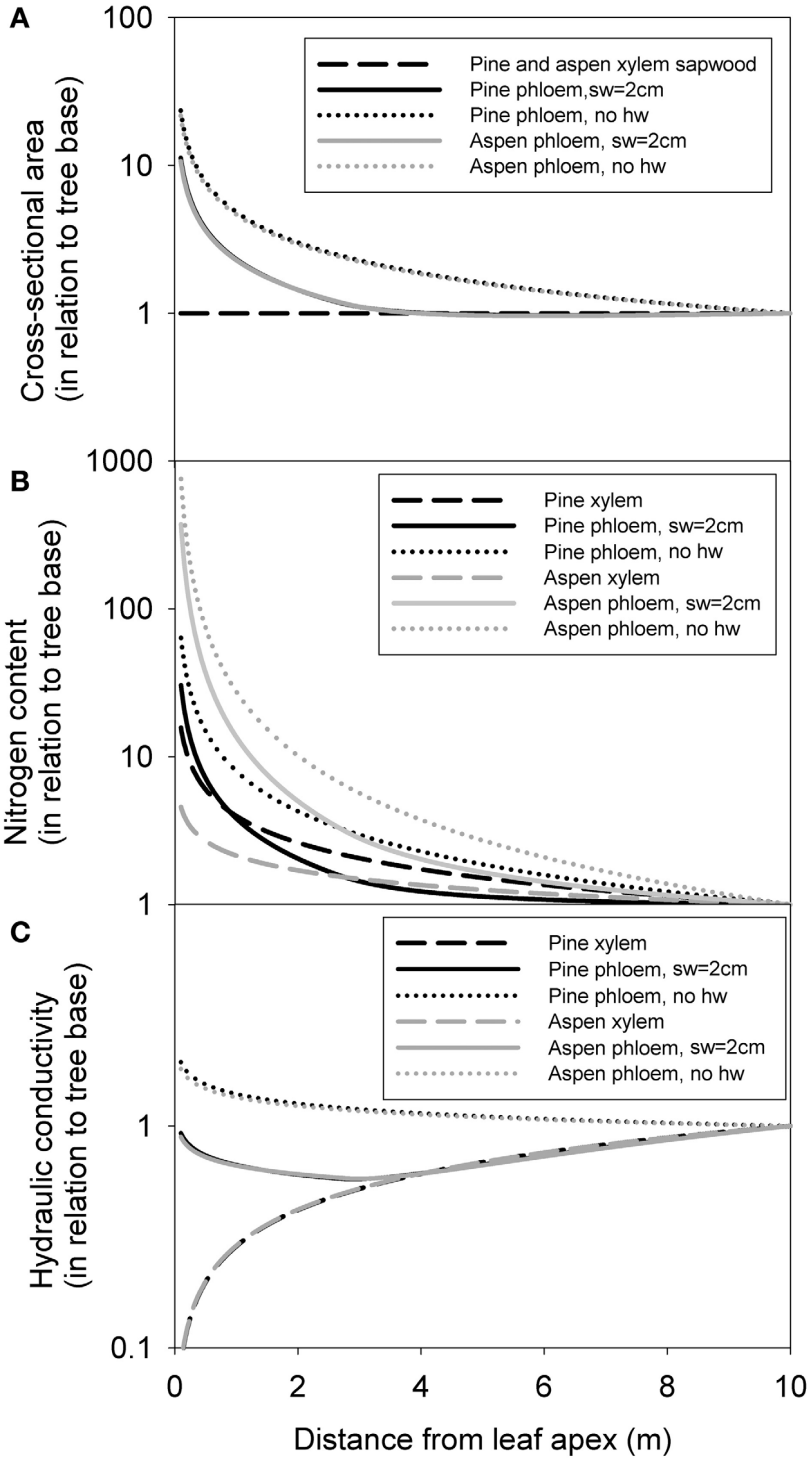
**The axial distribution of xylem sapwood and phloem cross-sectional area (A), nitrogen content (B) and hydraulic conductivity in a 10-m tree (C).** Values are expressed in relation to tree base in each case. “sw = 2 cm” refers to a scenario where maximum sapwood depth was assumed to be 2 cm, and “no hw” refers to a scenario of no heartwood formation, i.e., all of the xylem was assumed to be conducting.

Within a 10 m tree (taken as an example here) phloem cross-section (and volume) was distributed very much toward the apex, whereas xylem sapwood cross-section was evenly distributed axially, following from our pipe model assumption (Figure 7A). Xylem and phloem nitrogen content were more concentrated toward the apex (Figure 7B), but this relation was much stronger for the phloem, especially for aspen. Xylem conductivity was more concentrated (*k*_*x*_α *x*^0.5^) toward the base, whereas phloem conductivity was concentrated toward the apex when no heartwood formation was assumed (Figure 7C). Assuming maximum sapwood depth to be 2 cm caused phloem conductance to be distributed more evenly within the transport axis. The axial distribution of xylem and phloem properties was very similar in pine and aspen for cross-sectional area and conductance, but differed greatly for nitrogen content.

#### Simulations for the pressure and sugar gradients within the xylem and phloem, simulation 1

**Figure 8:**
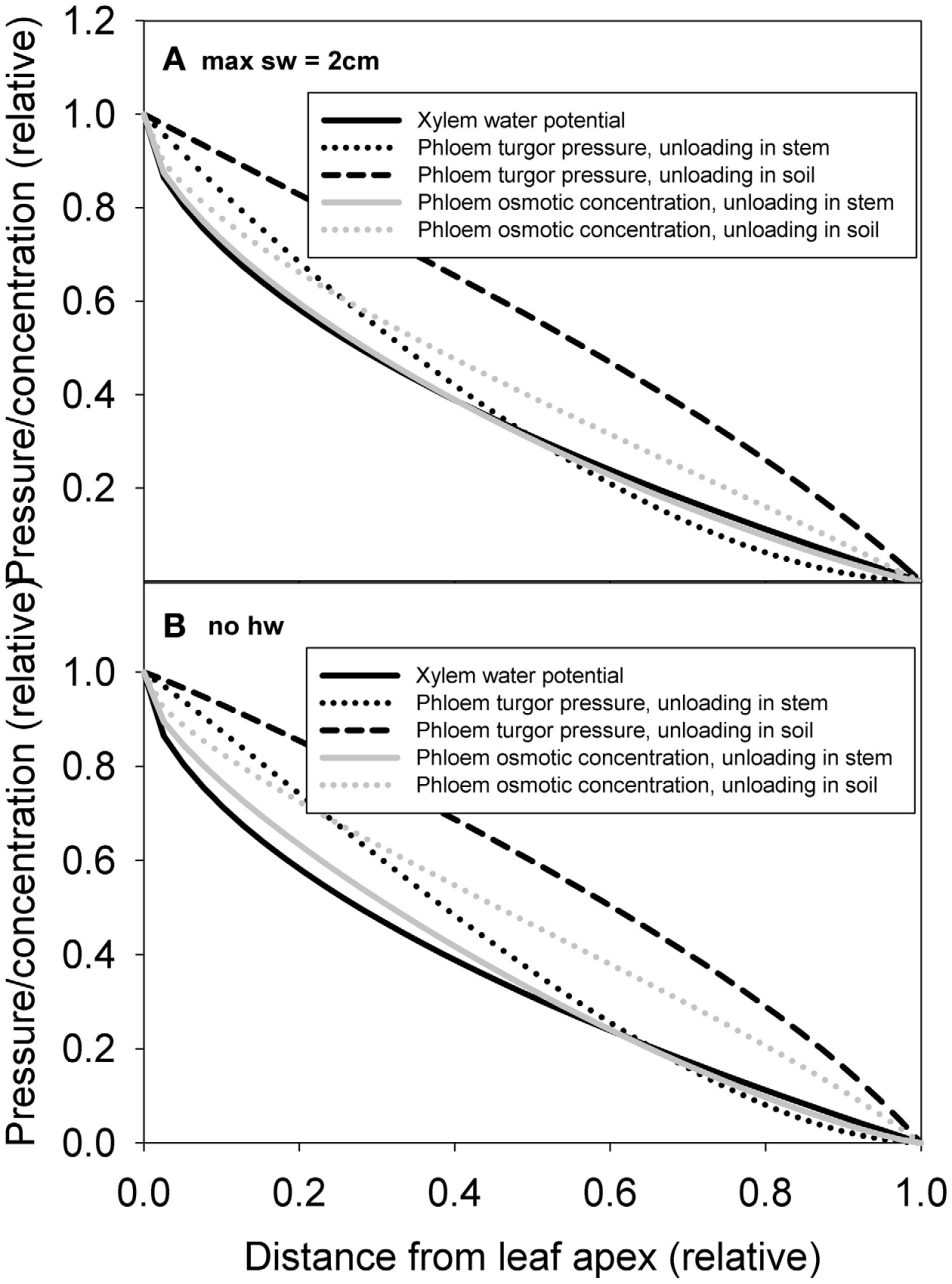
**Simulated xylem water potential, phloem turgor pressure and phloem osmotic concentration axial profiles for cases of phloem unloading in sink and phloem unloading along the stem for pine with an assumption of maximum sapwood depth of 2 cm (A) and no heartwood formation (B)**.

The xylem pressure (water potential) drop was predicted to occur more steeply close to the apex, while phloem pressure drop was predicted to occur more at the tree base in (Figures 8A,B), particularly when phloem unloading occurred in the soil. Phloem pressure gradients were sensitive to heartwood assumptions. In the absence of heartwood formation, phloem hydraulic conductivity was more concentrated toward the apex (see Figure 7C), which resulted in the phloem turgor pressure drop to concentrate more toward the base of the tree (Figure 8B). Phloem osmotic concentration gradient, which results from the interplay between both xylem and phloem transport properties, was predicted to be more evenly distributed over the transport axes. The normalized pressure and concentration gradients shown in the figure were not very sensitive to parameterization of the model, but the absolute values naturally were (not shown).

#### Simulations for “optimal” allocation of axial phloem transport capacity, simulation 2

When all of the sugar unloading occurred in the roots, the lowest turgor pressure difference between source and sink was obtained when phloem cross-sectional area scaled by *A*_*p*_α *x*^−0.17^ (*x* is distance from apex). When phloem unloading was made to occur evenly within the stem, a scaling relation of *A*_*p*_α *x*^−0.33^yielded the lowest turgor pressure difference. Importantly, the optimal axial allocation of phloem tissue predicted by the model was never as large as in the scaling results from the measurements, i.e., *A*_*p*_α *x*^−0.69^ for pine and *A*_*p*_α *x*^−0.67^ for aspen for a 10 m tree without heartwood and *A*_*p*_α *x*^−0.45^ for both pine and aspen when a maximum sapwood depth of 2 cm was assumed.

#### Simulated whole tree phloem turgor pressure differences, simulation 3

**Figure 9:**
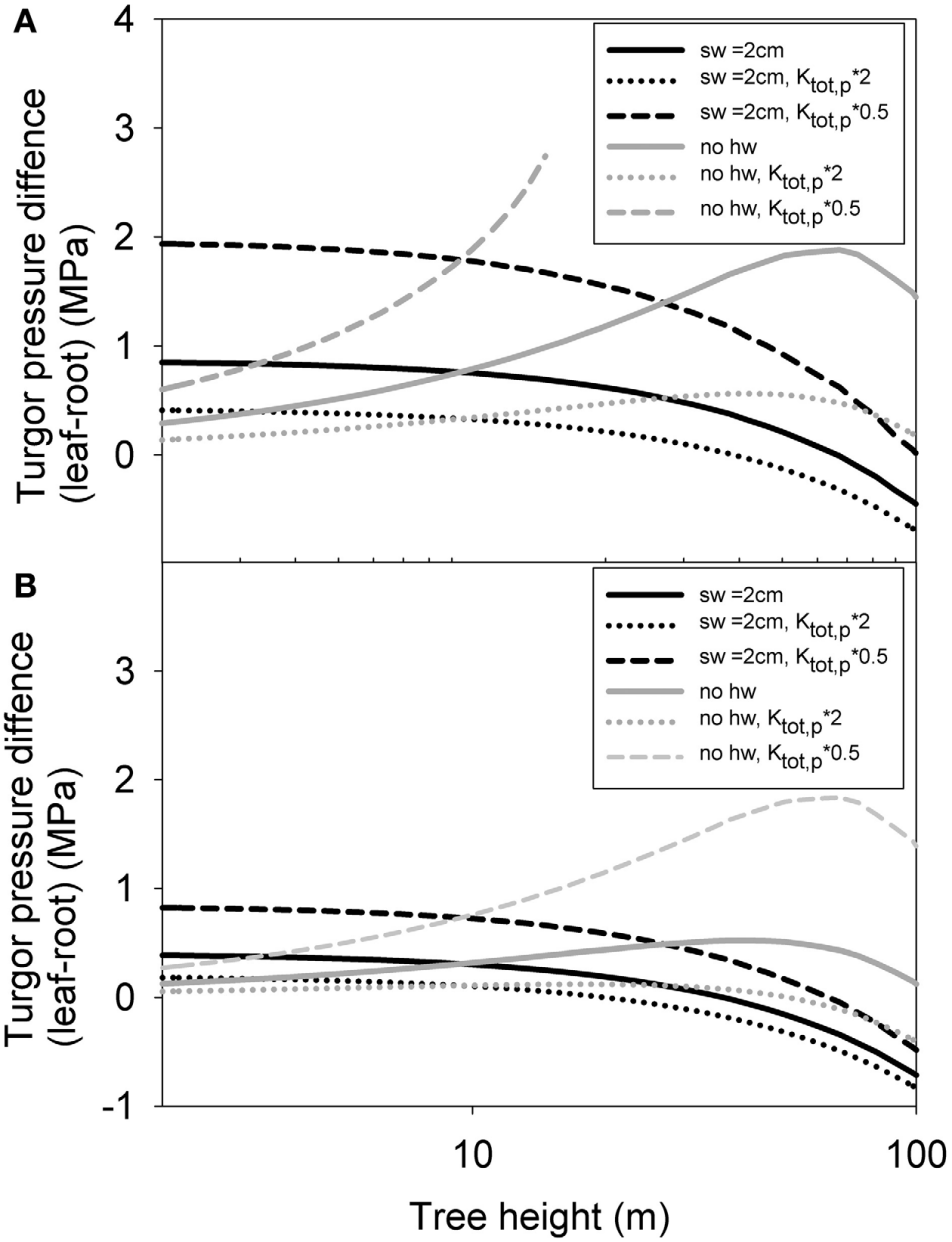
**Simulated turgor pressure difference between leaf and root phloem as a function of tree height with varying parameterization for a case where all phloem unloading occurs in the root (A) and evenly along the stem (B) for pine.** “sw = 2 cm” refers to a scenario where maximum sapwood depth was assumed to be 2 cm, and “no hw” refers to a scenario of no heartwood formation, i.e., all of the xylem was assumed to be conducting. “*K*_*p*, tot*_2,” and “*K*_*p*, tot*_0.5” refer cases where the absolute phloem conductance has been multiplied by a factor of 2 and 0.5, respectively.

Finally, in simulation 3, we analyzed how the whole tree level turgor pressure difference varies as a function of tree height using the predicted structural scaling of whole tree xylem and phloem hydraulic conductance. Phloem turgor pressure was predicted generally to increase slightly with increases in tree height when no heartwood formation was assumed, and to decrease slightly when maximum sapwood depth was limited to 2 cm (Figure 9). As the actual amount of sapwood can be predicted to lie in between these extreme scenarios, the turgor pressure differences between the leaves and roots could thus be expected to remain rather stable with increases in tree height. Phloem became unable to transport all of the assimilated sugars in trees larger than 15 m only in the case of low initial phloem conductivity and the assumption of no heartwood formation. In this case phloem sap viscosity experienced a sharp build up preventing an increase in the phloem transport despite an increase in the turgor pressure gradient. The increase in turgor pressure difference with increasing tree size was more pronounced when sugar unloading occurred exclusively in the roots (Figure 9A) in comparison to phloem unloading occurring evenly along the stem (Figure 9B). In many of the cases presented, phloem turgor pressure difference increased with increasing tree height for small trees, but then started to decline again. This was due to gravity which started become important for taller tree. Gravity aids phloem transport while decreasing the capacity of the xylem to transport water to the leaves. According to the isohydric scenario presented here, the decrease in xylem transport led to lower leaf exchange rates and thus also for a smaller transport need for the phloem. Increase in the initial value for phloem conductivity decreased the turgor pressure gradient for all tree sizes, as would be expected. Importantly, the turgor pressure difference between the leaves and roots required to drive the phloem transport of the assimilated sugars was predicted not to increase linearly with increases in tree height.

Pine was used as an example species in all of the simulations done with the xylem and phloem transport model, but the corresponding simulations for at least aspen would yield similar results as the scaling relations for the xylem and phloem volumes and hydraulic conductances are quite similar amongst the species (see Figure 4 and Table 4).

## Discussion

The equations constructed in this study make it possible to estimate whole tree level xylem and phloem properties (volume, hydraulic conductivity, nitrogen content). The equations require relations for xylem and phloem thicknesses/cross-sectional areas, conduit sizes and densities, and the nitrogen content across stem/branch samples of different diameters, and on the shape of the stem taper. Predictions can be made on how whole tree level properties scale with tree size assuming that the measured relationships do not change with tree height. This was supported by the data presented here on trees that varied in size measured for four different species. Phloem, bark, and xylem cross-sectional areas, and nitrogen content could be predicted with minimal deviation from stem/branch diameter alone using allometric, i.e., power law, relationships. The approach presented here can also be connected to functional-structural tree models that often provide detailed description of tree axes and their dimensions (e.g., Sievänen et al., 2000).

Phloem volume and nitrogen content were predicted to be concentrated heavily toward the tree apex, in contrast to the xylem, whose properties were more evenly distributed within a tree (Figure 7). Partially the latter was due to the pipe model assumption for the xylem. However, the pipe model assumption has been shown to work quite well for all the species analyzed in the measurements (Kaufmann and Troendle, 1981; Ilomaki et al., 2003; Kantola and Mäkelä, 2004; Berninger et al., 2005). Also phloem transport capacity (hydraulic conductance) was concentrated more toward the apex, especially if heartwood formation was limited. In contrast, xylem conductance was concentrated toward the base. In both cases the translocation capacity is thus largest closest to the source of the principal transported substance. Our measurements on the axial profile of the phloem thickness and cross-sectional area confirm the findings of Quilhó et al. (2000) who found that the thickness of conducting phloem cells is approximately constant along the stem height, while the cross-sectional area of water conducting xylem increases clearly from stem top toward base. The highly uneven axial distribution of nitrogen in both the xylem and phloem (Figure 3) signifies that nitrogen sampling must be done from varying branch sizes and/or tree heights in order to get an appropriate estimate for whole tree level xylem and phloem nitrogen content. For example, if nitrogen content sampling was done exclusively from larger stem and branch parts, then the total amount of nitrogen allocated to the vascular tissues would be grossly underestimated. This result has also direct implications to forest management where bioenergy harvesting is becoming more popular with the need for boosting the use of renewable energy sources. Our results imply that removal of distal parts of the crown from the growing site will deplete the ecosystem nitrogen pool as efficiently as the removal of leaves.

The relations between xylem sapwood and phloem volumes and conductances at the whole tree level were found to be sensitive to the assumption made about sapwood turnover to heartwood. When no heartwood formation was assumed, whole phloem conductance could not keep up with xylem conductance with increase in tree height. However, when a maximum sapwood radius of 2 cm was assumed, whole tree xylem and phloem conductances were predicted to change at approximately the same rates with tree growth, and xylem sapwood to phloem ratio was predicted to saturate approximately to a value of 10 (Figure 4A). This is approximately the same ratio as Hölttä et al. (2009) predicted based on the ratio of typical water to CO_2_ exchange rate the leaves and ranges of xylem and phloem pressure gradients typically observed.

It seems clear that the xylem and phloem become increasingly larger sinks of nitrogen in relation to foliage with increases in tree height, and that the nitrogen requirements of the vascular tissues could be a major limiting factor to tree growth in the Boreal region. In trees larger than ~10 m (Figures 4C,D; the exact height was depend on the species and the assumptions made about heartwood), xylem had the highest nitrogen content. Also some previous studies have reported large amounts of nitrogen in the stems of large trees (e.g., Helmisaari, 1995). Aspen had a larger proportion of nitrogen in the phloem in comparison to xylem and leaf than pine. Also the proportion of nitrogen in the xylem in comparison to the leaves was smaller in aspen in relation to pine. The case of nitrogen allocation between phloem and foliage is particularly interesting as there is a clear tradeoff between the nitrogen used to assimilation or assimilate transport. Already Mooney and Gulmon (1979) and Field (1983) suggested on theoretical grounds that optimal nitrogen allocation within tree crowns should yield constant photosynthetic nitrogen use efficiency. However, such distribution has rarely been found, probably owing to various other factors that influence photosynthetic production rate of foliage in the crown apart from nitrogen (e.g., Posada et al., 2012). In reality, the proportion of nitrogen allocated to the leaves could decline even more strongly with height than our analysis suggests as the leaf to sapwood ratio typically decreases with increases in tree height (e.g., McDowell et al., 2002a,b), which was not taken into account in our analysis. However, not all of the nitrogen found in the xylem and phloem is necessarily bound to the tissue structure, but it could also be in temporary storage there (Wetzel et al., 1989). Our study was conducted in the boreal environment where soil water availability is hardly ever restricting tree function and growth, while nitrogen is the main resource limitation for tree growth. It would be interesting to see if the allometric relations observed here diverge if trees from different environments would be added to the comparison.

The phloem transport capacity was predicted to decline more strongly than the xylem transport capacity when a tree grows in height, although the scenario of rapid xylem sapwood to heartwood turnover led to the ratio of phloem to xylem to phloem to stabilize at tree heights larger than 10 m. Theoretically, xylem and phloem transport conductances should scale almost equally with growth in height, if the ratios between water and carbon exchange and the driving forces for xylem and phloem transport are to be maintained. How is it then possible that leaf specific phloem transport capacity will decrease more in proportion to xylem transport capacity? We can hypothesize several explanations for this; (A) Gravity will increase the flow rate in the phloem and decrease the flow rate in the xylem for a given pressure gradient with increasing tree height. (B) A large proportion of the photosynthates might be consumed close to the apex in tall trees, so that phloem conductance can be allowed to decline at lower heights in the tree. This is in contrast to the xylem where practically all of the water is transported all the way from soil to the foliage. (C) Trees compensate for the decreased leaf area specific phloem conductance by increasing the turgor and osmotic pressure gradient in the phloem as a tree grows in height. Our simulations with the coupled xylem and phloem transport model revealed that the factors given above could explain the functioning of phloem transport by the Münch pressure flow hypothesis with increasing tree height even when phloem conductance decreases slightly in relation to xylem conductance with increasing tree height. The simulations also revealed that in most of the scenarios explored the turgor pressure difference between the leaves and roots remained rather constant with increases in tree height. This result is line with a recent review (Turgeon, 2010) which stated that there are indications that the turgor pressure differences between the sources and sink would not increase with tree size.

The xylem and phloem transport model also predicted that concentrating phloem volume more toward the leaf apex yielded lower turgor pressure difference between leaves and roots, especially if part of the sugars transported in the phloem are utilized along the stem. However, the actual increase in phloem volume toward the apex (based on the measurements and the scaling presented in this study) was found to be even larger than that predicted by the transport model. Also the within tree gradients of turgor pressure and osmotic concentration can be predicted from the axial distribution of xylem sapwood and phloem volumes and area-specific conductivities using a transport model (Figure 8). One can hypothesize a feedback loop between local pressure and osmotic concentration mediated by xylem and phloem conductances, and the local growth rate of new xylem and phloem tissue. This feedback loop, spanning several growing seasons, could be explained by the direct link between cell division, expansion, and cell wall synthesis on the local water and carbon status (e.g., De Schepper and Steppe, 2010; Hölttä et al., 2010; Pantin et al., 2012) providing a structural-functional automata for vascular development in trees.

Xylem conductance also decreases with growth in height, but not nearly as sharply as phloem conductance. Xylem conductance decreases at the rate of square root of tree height, which stems from two simple empirical observations: the cross-sectional area of xylem sapwood over branching is conserved (Shinozaki et al., 1964) and the increase in xylem conduit radius with tree height (*r*_*x*_α *x*^1/4^) (West et al., 1999). Various findings also support the notion that tree transpiration and photosynthesis rates decrease with tree height as either stomatal conductance and/or leaf to sapwood area decrease (McDowell et al., 2002a,b, 2005; Martínez-Vilalta et al., 2007). The strong decrease of phloem conducting capacity toward the tree base stems from the observation that the phloem width does not change very much from tree top to the base (see also Quilhó et al., 2000). One possible explanation for this is that it follows from the limitations of cambium activity. Unlike the sapwood, which accumulates over several years, phloem apparently needs to be renewed practically yearly (Ewers, 1982). Secondary growth results from rate of cell division and their subsequent enlargement. These are constrained by the length of the growth period and temperature during the period but also by the water status and sugar supply to the growth location (e.g., De Schepper and Steppe, 2010; Hölttä et al., 2010). While it seems that the vigor of the tree may influence the extension of growth period (Rathgeber et al., 2011) it is ultimately influenced by the duration of the favorable conditions, particularly in boreal environment. The water status and available sugars are influenced by the tree size such that there is less foliage, and presumably sugars, relative to stem the bigger the trees. For these reasons, the annual width of phloem growth could be limited, and although the relatively constant phloem width in axial direction means that phloem width increases relative to yearly tree ring width from top to base, it is not able to compensate for the different functional longevities of the tissues. The increasing girth of the trees, however, will help to balance the difference. We could even hypothesize that the need to balance the amounts of phloem and xylem tissue would be behind the stem diameter growth and sapwood turnover. With a given sapwood requirement, its higher turnover would necessarily mean faster thickness growth, which would have a large impact also on resource allocation and tree development (Nikinmaa, 1992). All these functional-structural interactions impose strong boundary conditions for the tree development and function.

## Conclusions

The study showed that important understanding of whole tree functions can be gained by dimensional analysis across tree axes. The observed regularities in xylem and phloem structure, and the Münch- pressure flow hypothesis seem to provide a feasible explanation of phloem transport even in the largest trees. Sapwood turnover to heartwood seems to have an important functional role in affecting the scaling relations for xylem and phloem hydraulic conductances and nitrogen allocation. Xylem and phloem tissues are clearly a larger sink of nitrogen than the foliage as trees grow in height becoming an important and an often overlooked factor in the forest nitrogen cycle particularly in the nitrogen limited boreal forest where the slow nitrogen turnover rate is often the reason for growth limitation.

### Conflict of interest statement

The authors declare that the research was conducted in the absence of any commercial or financial relationships that could be construed as a potential conflict of interest.

## Appendix

### A1. derivation of the analytic scaling functions for xylem and phloem properties as a function of tree size

The number of furcations at distance of *x* from the leaf apex is (assuming pipe model relations, i.e., *nAx* is preserved)

(A1) $$n(x)=\frac{n_{\text{ref}}A_{x,\text{ ref}}}{A_{x}(x)}=\frac{n_{\text{ref}}\alpha_{1}\gamma^{\beta_{1}}L^{\delta \beta_{1}}}{\alpha_{1}\gamma^{\beta_{1}}x^{\delta \beta_{1}}}=\frac{L^{\delta \beta_{1}}}{x^{\delta \beta_{1}}}={\left(\frac{L}{x}\right)}^{\delta \beta_{1}}$$

where *n*_ref_ is the number of furcations at the base of tree (*n*_ref_ = 1) is and *L* is total tree height.

Total amount of xylem in a tree as a function of tree height (starting from a distance *L*_0_ away from leaf apex) is

(A2) $$\begin{array}{l} V_{x,\text{ tot}}=\int_{0}^{L}nA_{x}dx=\int_{0}^{L}{\left(\frac{L}{x}\right)}^{\delta \beta_{1}}\alpha_{1}\gamma^{\beta_{1}}x^{\delta \beta_{1}}dx\\ \;=\int_{0}^{L}L^{\delta \beta_{1}}\alpha_{1}\gamma^{\beta_{1}}x^{\delta \beta_{1}}x^{-\delta \beta_{1}}dx=\int_{0}^{L}L^{\delta \beta_{1}}\alpha_{1}\gamma^{\beta_{1}}dx=\\ \;=L^{\delta \beta_{1}}\alpha_{1}\gamma^{\beta_{1}}L=\alpha_{1}\gamma^{\beta_{1}}L^{\delta \beta_{1}+1}-\alpha_{1}\gamma^{\beta_{1}}L_{0}^{\delta \beta_{1}+1} \end{array}$$

If the distance *L*_0_ is more than zero (e.g., if measurements only exist starting from a distance *L*_0_ from the apex as in our measurements) and if the xylem properties are assumed to remain constant between distance 0 and *L*_0_, the amount xylem between 0 and *L*_0_ (0.1 m in this case) must be added to the sum. Xylem properties were assumed to be constant (the same as at a distance *L*_0_ from the apex = in this range. The amount of xylem between 0 and 0.1 m is

(A3) $$\begin{array}{l} V_{x,\;0-L_{0}}=nA_{x}dx={\left(\frac{L}{L_{0}}\right)}^{\delta \beta_{1}}\alpha_{1}\gamma^{\beta_{1}}L_{0}^{\delta \beta_{1}}L_{0}\\ \;={\left(\frac{L}{0.1\text{m}}\right)}^{\delta \beta_{1}}\alpha_{1}\gamma^{\beta_{1}}0.1{\text{m}}^{\delta \beta_{1}}*0.1\text{m} \end{array}$$

Total amount of phloem in tree as a function of tree height is

(A4) $$\begin{array}{l} V_{p,\text{ tot}}=\text{​}\int_{L_{0}}^{L}nA_{p}dx=\int_{L_{0}}^{L}{\left(\frac{L}{x}\right)}^{\delta \beta_{1}}\alpha_{2}\gamma^{\beta_{2}}x^{\delta \beta_{2}}dx\\ \;=\int_{L_{0}}^{L}L^{\delta \beta_{1}}\alpha_{2}\gamma^{\beta_{2}}x^{\delta \beta_{2}}x^{-\delta \beta_{1}}dx=\text{​}\int_{L_{0}}^{L}L^{\delta \beta_{1}}\alpha_{2}\gamma^{\beta_{2}}x^{\delta \beta_{2}-\delta \beta_{1}}dx=\\ \text{ ​}=\frac{L^{\delta \beta_{1}}\alpha_{2}\gamma^{\beta_{2}}}{\delta \beta_{2}-\delta \beta_{1}+1}\left(L^{\delta \beta_{2}-\delta \beta_{1}+1}-L_{0}^{\delta \beta_{2}-\delta \beta_{1}+1}\right)\\ \;=\frac{L^{\delta \beta_{1}}\alpha_{2}\gamma^{\beta_{2}}}{\delta \beta_{2}-\delta \beta_{1}+1}\text{​}\left(L^{\delta \beta_{2}-\delta \beta_{1}+1}-L_{0}^{\delta \beta_{2}-\delta \beta_{1}+1}\right) \end{array}$$

where *L*_0_ is the height (distance from apex) from which the integration is started from. The integration could not be started from *L*_0_ = 0 as the phloem volume approaches infinity when *L*_0_ approaches 0.

Also, the amount of phloem between 0 and *L*_0_ must be added to the sum. Phloem properties were assumed to be constant in this range. The amount of phloem between 0 and 0.1 m is

(A5) $$\begin{array}{l} V_{p,\;0-L_{0}}=nA_{p}dx={\left(\frac{L}{L_{0}}\right)}^{\delta \beta_{1}}\alpha_{2}\gamma^{\beta_{2}}L_{0}^{\delta \beta_{2}}L_{0}\\ \;={\left(\frac{L}{0.1\text{m}}\right)}^{\delta \beta_{1}}\alpha_{2}\gamma^{\beta_{2}}\text{0}.{\text{1m}}^{\delta \beta_{2}}*0.1\text{m} \end{array}$$

Total amount of nitrogen in xylem as a function of tree height is

(A6) $$\begin{array}{l} \;N_{x,\text{ tot}}=\int_{L_{0}}^{L}nA_{x}\rho_{N,\;x}\rho_{x}dx\\ \;=\int_{L_{0}}^{L}{\left(\frac{L}{x}\right)}^{\delta \beta_{1}}\alpha_{1}\gamma^{\beta_{1}}x^{\delta \beta_{1}}\alpha_{3}\gamma^{\beta_{3}}x^{\delta \beta_{3}}\rho_{x}dx\\ \;=\int_{L_{0}}^{L}L^{\delta \beta_{1}}\alpha_{1}\gamma^{\beta_{1}}\alpha_{3}\gamma^{\beta_{3}}x^{\delta \beta_{1}}x^{-\delta \beta_{1}}x^{\delta \beta_{3}}\rho_{x}dx=\\ \int_{L_{0}}^{L}L^{\delta \beta_{1}}\alpha_{1}\gamma^{\beta_{1}}\alpha_{3}\gamma^{\beta_{3}}x^{\delta \beta_{3}}\rho_{x}dx=\frac{L^{\delta \beta_{1}}\alpha_{1}\gamma^{\beta_{1}}\alpha_{3}\gamma^{\beta_{3}}\rho_{x}L^{\delta \beta_{3}+1}}{\delta \beta_{3}+1}\\ \text{​ }=\frac{\alpha_{1}\gamma^{\beta_{1}}\alpha_{3}\gamma^{\beta_{3}}\rho_{x}}{\delta \beta_{3}+1}L^{\delta \beta_{1}\text{+}\delta \beta_{3}+1}-\frac{\alpha_{1}\gamma^{\beta_{1}}\alpha_{3}\gamma^{\beta_{3}}\rho_{x}}{\delta \beta_{3}+1}L_{0}^{\delta \beta_{1}\text{+}\delta \beta_{3}+1} \end{array}$$

where ρ_*x*_ is the density of the xylem tissue (gm^−3^). We assume ρ_*N*_ to be 0.5 * 10^−6^ gm^−3^.

Also, the amount nitrogen in the xylem between 0 and *L*_0_ must be added to the sum. Phloem properties were assumed to be constant in this range. The amount of phloem between 0 and 1 m is

(A7) $$\begin{array}{l} N_{x,\;0-L_{0}}=nA_{x}\rho_{x}\rho_{x}dx={\left(\frac{L}{L_{0}}\right)}^{\delta \beta_{1}}\alpha_{1}\gamma^{\beta_{1}}L_{0}^{\delta \beta_{1}}\alpha_{3}\gamma^{\beta_{3}}\rho_{N}L_{0}^{\delta \beta_{3}}L_{0}\\ \;=\text{​}{\left(\frac{L}{1\text{m}}\right)}^{\delta \beta_{1}}\text{​​}\alpha_{1}\gamma^{\beta_{1}}\text{​}{\left(\text{1m}\right)}^{\delta \beta_{1}}\alpha_{3}\gamma^{\beta_{3}}\rho_{N}{\text{1m}}^{\delta \beta_{3}}*1\text{m}\;\;\;\; \end{array}$$

Total amount of nitrogen in phloem as a function of tree height is

(A8) $$\begin{array}{l} N_{p,\text{ tot}}=\int_{0}^{L}nA_{p}\rho_{p}\rho_{N}dx=\int_{0}^{L}{\left(\frac{L}{x}\right)}^{\delta \beta_{1}}\alpha_{2}\gamma^{\beta_{2}}x^{\delta \beta_{2}}\alpha_{4}\gamma^{\beta_{4}}\rho_{N}x^{\delta \beta_{4}}dx\\ \;=\int_{0}^{L}L^{\delta \beta_{1}}\alpha_{2}\gamma^{\beta_{2}}\alpha_{4}\gamma^{\beta_{4}}\rho_{N}x^{\delta \beta_{2}}x^{-\delta \beta_{1}}x^{\delta \beta_{4}}dx=\\ \;=\int_{0}^{L}L^{\delta \beta_{1}}\alpha_{2}\gamma^{\beta_{2}}\alpha_{4}\gamma^{\beta_{4}}\rho_{N}x^{\delta \beta_{4}+\delta \beta_{2}-\delta \beta_{1}}dx\\ \;=\frac{L^{\delta \beta_{1}}\alpha_{2}\gamma^{\beta_{2}}\alpha_{4}\rho_{N}\gamma^{\beta_{4}}}{\delta \beta_{4}+\delta \beta_{2}-\delta \beta_{1}+1}\left(L^{\delta \beta_{4}+\delta \beta_{2}-\delta \beta_{1}+1}-L_{0}^{\delta \beta_{4}+\delta \beta_{2}-\delta \beta_{1}+1}\right) \end{array}$$

ρ_*N*_ is the density of the phloem tissue (g m^−3^). We assume ρ_*N*_ to be 0.5 * 10^−6^ gm^−3^, i.e., the same as for the xylem tissue.

Also, the amount nitrogen in the phloem between 0 and *L*_0_ must be added to the sum. Phloem properties were assumed to be constant in this range. The amount of phloem between 0 and 1 m is

(A9) $$\begin{array}{l} N_{p,\;0-L_{0}}=nA_{p}\rho_{p}\rho_{N}dx={\left(\frac{L}{L_{0}}\right)}^{\delta \beta_{1}}\alpha_{2}\gamma^{\beta_{2}}{\text{L}}_{0}^{\delta \beta_{2}}\alpha_{4}\gamma^{\beta_{4}}\rho_{N}L_{0}^{\delta \beta_{4}}L_{0}\\ \;=\text{​}{\left(\frac{L}{1\text{m}}\right)}^{\delta \beta_{1}}\text{​​}\alpha_{2}\gamma^{\beta_{2}}\text{​}{\left(1\text{m}\right)}^{\delta \beta_{2}}\alpha_{4}\gamma^{\beta_{4}}\rho_{N}1{\text{m}}^{\delta \beta_{4}}*1\text{m} \end{array}$$

Total hydraulic conductance of the xylem is

(A10) $$\begin{array}{l} K_{x,\text{ tot}}^{-1}=\int_{0}^{L}{\left(nA_{x}\rho_{c,x}r_{x}^{2}\right)}^{-1}dx\\ \;={\int_{0}^{L}\left({\left(\frac{L}{x}\right)}^{\delta \beta_{1}}\alpha_{1}\gamma^{\beta_{1}}x^{\delta \beta_{1}}\alpha_{7}\gamma^{\beta_{7}}x^{\delta \beta_{7}}\alpha_{5}^{2}\gamma^{2\beta_{5}}x^{2\delta \beta_{5}}\right)}^{-1}dx\\ \;={\int_{0}^{L}\left(L^{\delta \beta_{1}}\alpha_{1}\alpha_{7}\alpha_{5}^{2}\gamma^{\beta_{1}+\beta_{7}+2\beta_{5}}\right)}^{-1}x^{-\delta \beta_{7}-2\delta \beta_{5}}dx\\ \;={\left(L^{\delta \beta_{1}}\alpha_{1}\alpha_{7}\alpha_{5}^{2}\gamma^{\beta_{1}+\beta_{7}+2\beta_{5}}\right)}^{-1}\\ \text{​ }\frac{1}{-\delta \beta_{7}-2\delta \beta_{5}+1}\left(L^{-\delta \beta_{7}-2\delta \beta_{5}+1}-L_{0}^{-\delta \beta_{7}-2\delta \beta_{5}+1}\right) \end{array}$$

Also, the xylem resistance between 0 and *L*_0_ must be added to the sum. Xylem properties were assumed to be constant in this range. Xylem conductance between 0 and *L*_0_ is

(A11) $$\begin{array}{l} K_{x,\;0-L_{0}}^{-1}={\left(nA_{x}\rho_{c,\;x}r_{x}^{2}\right)}^{-1}dx\\ \;={\left({\left(\frac{L}{L_{0}}\right)}^{\delta \beta_{1}}\alpha_{1}\gamma^{\beta_{1}}L_{0}^{\delta \beta_{1}}\alpha_{7}\gamma^{\beta_{7}}L_{0}^{\delta \beta_{7}}\alpha_{5}^{2}\gamma^{\text{2}\beta_{5}}L_{0}^{\text{2}\delta \beta_{5}}\right)}^{-1}L_{0}\\ \;={\left({\left(\frac{L}{L_{0}}\right)}^{\delta \beta_{1}}\alpha_{1}\alpha_{7}\alpha_{5}^{2}\gamma^{\beta_{1}\text{+}\beta_{7}\text{+2}\beta_{5}}L_{0}^{\delta \beta_{1}+\delta \beta_{7}\text{+2}\delta \beta_{5}}\right)}^{-1}L_{0} \end{array}$$

Total hydraulic conductance of the phloem is

(A12) $$\begin{array}{l} K_{p,\text{ tot}}^{-1}=\text{​}\int_{0}^{L}{\left(nA_{p}\rho_{c,\;P}r_{p}^{2}\right)}^{-1}dx\\ \;=\text{​}\int_{0}^{L}{\left(L^{\delta \beta_{1}}\alpha_{2}\alpha_{8}\alpha_{6}^{2}\gamma^{\beta_{2}\text{+}\beta_{8}+2\beta_{6}}\right)}^{-1}x^{-\delta \beta_{2}+\delta \beta_{1}-\delta \beta_{8}-2\delta \beta_{6}}dx=\\ \;=\text{​}{\left(L^{\delta \beta_{1}}\alpha_{2}\alpha_{8}\alpha_{6}^{2}\gamma^{\beta_{2}+\beta_{8}+2\beta_{6}}\right)}^{-1}\text{​}\frac{1}{-\delta \beta_{2}+\delta \beta_{1}-\delta \beta_{8}-2\delta \beta_{6}+1}\\ \text{ ​}\left(L^{-\delta \beta_{2}+\delta \beta_{1}-\delta \beta_{8}-2\delta \beta_{6}+1}-L_{0}^{-\delta \beta_{2}+\delta \beta_{1}-\delta \beta_{8}-2\delta \beta_{6}+1}\right) \end{array}$$

Also, the amount phloem between 0 and *L*_0_ must be added to the sum. Phloem properties were assumed to be constant in this range. The amount of phloem between 0 and *L*_0_ is

(A13) $$\begin{array}{l} K_{p,\;0-L_{0}}^{-1}={\left(nA_{p}\rho_{c,\;P}r_{p}^{2}\right)}^{-1}dx\\ \;={\left({\left(\frac{L}{L_{0}}\right)}^{\delta \beta_{1}}\alpha_{2}\gamma^{\beta_{2}}L_{0}^{\delta \beta_{2}}\alpha_{8}\gamma^{\beta_{8}}L_{0}^{\delta \beta_{8}}\alpha_{6}^{2}\gamma^{\text{2}\beta_{6}}L_{0}^{2\delta \beta_{6}}\right)}^{-1}L_{0}\\ \;=\left({\left(\frac{L}{0.1\text{m}}\right)}^{\delta \beta_{1}}\alpha_{2}\alpha_{8}\alpha_{6}^{2}\gamma^{\beta_{2}+\beta_{8}+2\beta_{6}}{\left(0.1\text{m}\right)}^{\delta \beta_{2}+\delta \beta_{8}+2\delta \beta_{6}}\right)\text{​}-1*0.1\text{m} \end{array}$$

### Nitrogen in leaves

Assuming that leaf area scales with xylem sapwood cross-sectional area and that leaf nitrogen content is constant, total amount of nitrogen in leaves can be estimated to be

(A14) $$A_{\text{leaf}}=A_{x}*C_{ls}$$

where *C*_*ls*_ is the leaf to sapwood ratio

(A15) $$N_{\text{leaf},\text{ tot}}=A_{\text{leaf}}\rho_{\text{SLA}}\rho_{N,\text{ leaf}}=A_{x,\text{ ref}}C_{ls}\rho_{\text{leaf}}\rho_{N,\text{ leaf}}$$

In accordance with the pipe model prediction, xylem cross-sectional area equals total xylem volume divided by tree height. Total leaf nitrogen content can then be written as

(A16) $$N_{\text{leaf},\text{ tot}}=\frac{\alpha_{1}\gamma^{\beta_{1}}\left(L^{\delta \;\beta_{1}+1}-L_{0}^{\delta \beta_{1}+1}\right)}{L}C_{ls}\rho_{\text{SLA}}\rho_{N,\text{ leaf}}$$

The parameters used in the calculations are shown in Table 3. The values for leaf-to-sapwood area ratio, specific leaf area, and leaf nitrogen content were taken for pine from the Supplementary material in Martinez-Vilalta et al. (2009) in which these values are reported for the SMEAR II Hyytiälä research station in Hyytiälä Southern Finland, the same place from where most of the trees were taken for our measurements. Korhonen et al. (2013) reported a very similar value, 1.2%, for leaf nitrogen content of 1.2% at the same site. The corresponding values for aspen could not be obtained for the same site, and the values are taken studies of Niinemets (1997), and Hoffmann and Usoltsev (2002). Values for both pine and aspen are in line with values from metastudies containing several species: McDowell et al. (2002a,b) compiled data of leaf to sapwood area from 14 different studies containing conifer and broadleaved trees. The average of the species was ~2700 m^2^ (leaf area)/m^2^ (sapwood). Lusk et al. (2003) compiled data average for 23 evergreen angiosperm and 20 conifer populations from six sites in American continent and found an SLA of 150 g/m^2^ and nitrogen content of 1.14% on average. Reich et al. (1998) compiled data from 69 species from four functional groups (forbs, broad-leafed trees and shrubs, and needle-leafed conifers) in six sites in American continent and found an average SLA 140 g/m^2^ and nitrogen content 1.7%.

## A2. derivation of the scaling functions with heartwood included

In additional calculations, we also took into account that part of the xylem is heartwood. We assumed that there is a maximum sapwood radius *r*_*sw*, max_, and that any xylem tissue radial inwards to the maximum sapwood depth was heartwood. We assumed that heartwood did not contain any nitrogen and did not contribute to xylem conductance. We did not find analytical solutions to the scaling in the case of heartwood inclusion. Results shown are from the following numerical integration, using a numerical integration interval (*dx*) of 1 mm.

(A17) $$\begin{array}{l} A_{\text{sw}}=A_{x}-A_{\text{hw}}=\alpha_{1}\gamma^{\beta_{1}}x^{\delta \beta_{1}}-\pi {\left(r_{\text{xyl}}-r_{sw,\text{ max}}\right)}^{2}\\ \;=\alpha_{1}\gamma^{\beta_{1}}x^{\delta \beta_{1}}-\pi {\left(\alpha_{1}\gamma^{\beta_{1}}x^{\delta \beta_{1}}-r_{sw,\;\max}\right)}^{2}\text{if}r_{\text{xyl}}>r_{sw,\text{ max}} \end{array}$$

The number of furcations at distance of *x* from the leaf apex is then

(A18) $$n(x)=\frac{n_{\text{ref}}A_{\text{sw},\text{ ref}}}{A_{\text{sw}}(x)}=\frac{\left(\alpha_{1}\gamma^{\beta_{1}}L^{\delta \beta_{1}}-\pi {\left(r_{xyl}(L)-r_{\text{sw},\;\max}\right)}^{2}\right)}{\left(\alpha_{1}\gamma^{\beta_{1}}x^{\delta \beta_{1}}-\pi {\left(r_{xyl}(x)-r_{\text{sw},\;\max}\right)}^{2}\right)}$$

Total amount of xylem sapwood is then

(A19) $$\begin{array}{l} V_{x,\text{ tot}}=\int_{0}^{L}nA_{x}dx=\int_{0}^{L}\frac{\left(\alpha_{1}\gamma^{\beta_{1}}L^{\delta \beta_{1}}-\pi {\left(r_{\text{xyl}}(L)-r_{sw,\;\max}\right)}^{2}\right)}{\left(\alpha_{1}\gamma^{\beta_{1}}x^{\delta \beta_{1}}-\pi {\left(r_{\text{xyl}}(x)-r_{sw,\;\max}\right)}^{2}\right)}\\ \;\left(\alpha_{1}\gamma^{\beta_{1}}x^{\delta \beta_{1}}-\pi {\left(r_{\text{xyl}}(x)-r_{sw,\;\max}\right)}^{2}\right)dx\\ \;=\int_{0}^{L}\left(\alpha_{1}\gamma^{\beta_{1}}x^{\delta \beta_{1}}-\pi {\left(r_{\text{xyl}}(x)-r_{sw,\;\max}\right)}^{2}\right)dx \end{array}$$

Total amount of phloem is then

(A20) $$\begin{array}{l} V_{p,\text{ tot}}=\int_{0}^{L}nA_{p}dx\\ \;=\int_{0}^{L}\frac{\left(\alpha_{1}\gamma^{\beta_{1}}L^{\delta \beta_{1}}-\pi {\left(r_{xyl}(L)-r_{sw,\;\max}\right)}^{2}\right)}{\left(\alpha_{1}\gamma^{\beta_{1}}x^{\delta \beta_{1}}-\pi {\left(r_{xyl}(x)-r_{sw,\max}\right)}^{2}\right)}\alpha_{2}\gamma^{\beta_{2}}x^{\delta \beta_{2}}dx \end{array}$$

Total amount of nitrogen in the xylem is then

(A21) $$\begin{array}{l} N_{x,\text{ tot}}=\int_{0}^{L}nA_{x}\rho_{N,\;x}\rho_{x}dx\\ \;=\int_{0}^{L}\frac{\left(\alpha_{1}\gamma^{\beta_{1}}L^{\delta \beta_{1}}-\pi {\left(r_{\text{xyl}}(L)-r_{sw,\;\max}\right)}^{2}\right)}{\left(\alpha_{1}\gamma^{\beta_{1}}x^{\delta \beta_{1}}-\pi {\left(r_{\text{xyl}}(x)-r_{sw,\;\max}\right)}^{2}\right)}\\ \;\left(\alpha_{1}\gamma^{\beta_{1}}x^{\delta \beta_{1}}-\pi {\left(r_{\text{xyl}}(x)-r_{sw,\;\max}\right)}^{2}\right)\alpha_{3}\gamma^{\beta_{3}}x^{\delta \beta_{3}}\rho_{x}dx \end{array}$$

Total amount of nitrogen in the phloem is then

(A22) $$\begin{array}{l} N_{p,\text{ tot}}=\int_{0}^{L}nA_{p}\rho_{p}\rho_{N}dx\\ \;=\int_{0}^{L}\frac{\left(\alpha_{1}\gamma^{\beta_{1}}L^{\delta \beta_{1}}-\pi {\left(r_{\text{xyl}}(L)-r_{sw,\;\max}\right)}^{2}\right)}{\left(\alpha_{1}\gamma^{\beta_{1}}x^{\delta \beta_{1}}-\pi {\left(r_{\text{xyl}}(x)-r_{sw,\;\max}\right)}^{2}\right)}\alpha_{2}\gamma^{\beta_{2}}x^{\delta \beta_{2}}\alpha_{4}\gamma^{\beta_{4}}\rho_{N}x^{\delta \beta_{4}}dx \end{array}$$

Xylem hydraulic conductance is then

(A23) $$\begin{array}{l} K_{x,\text{ tot}}^{-1}=\int_{0}^{L}{\left(nA_{x}\rho_{c,\;x}r_{x}^{2}\right)}^{-1}dx=\\ \;=\int_{0}^{L}(\frac{\left(\alpha_{1}\gamma^{\beta_{1}}L^{\delta \beta_{1}}-\pi {\left(r_{\text{xyl}}(L)-r_{sw,\;\max}\right)}^{2}\right)}{\left(\alpha_{1}\gamma^{\beta_{1}}x^{\delta \beta_{1}}-\pi {\left(r_{\text{xyl}}(x)-r_{sw,\;\max}\right)}^{2}\right)}\\ \;{\left(\alpha_{1}\gamma^{\beta_{1}}x^{\delta \beta_{1}}-\pi {\left(r_{\text{xyl}}(x)-r_{sw,\;\max}\right)}^{2}\right)\alpha_{7}\alpha_{5}^{2}\gamma^{\beta_{7}+2\beta_{5}}x^{\delta \beta_{7}+2\delta \beta_{5}})}^{-1}dx \end{array}$$

Phloem hydraulic conductance is then

(A24) $$\begin{array}{l} K_{p,\text{ tot}}^{-1}=\int_{0}^{L}{\left(nA_{p}\rho_{c,\;P}r_{p}^{2}\right)}^{-1}dx\\ \;=\int_{0}^{L}(\frac{\left(\alpha_{1}\gamma^{\beta_{1}}L^{\delta \beta_{1}}-\pi {\left(r_{\text{xyl}}(L)-r_{sw,\;\max}\right)}^{2}\right)}{\left(\alpha_{1}\gamma^{\beta_{1}}x^{\delta \beta_{1}}-\pi {\left(r_{\text{xyl}}(x)-r_{sw,\;\max}\right)}^{2}\right)}\\ \;{\alpha_{2}\alpha_{8}\alpha_{6}^{2}\gamma^{\beta_{2}+\beta_{8}+2\beta_{6}}x^{\delta \beta_{2}+\delta \beta_{8}+2\delta \beta_{6}})}^{-1}dx \end{array}$$

Total leaf area is then proportional to sapwood, instead of whole xylem, cross-sectional area

(A25) $$A_{\text{leaf}}=A_{sx}*C_{ls}$$

## A3. derivation of the analytic scaling functions for the axial distribution of xylem and phloem properties for a given tree size

Axial distribution of xylem volume is

(A26) $$V_{x}(x)=nA_{x}={\left(\frac{L}{x}\right)}^{\delta \beta_{1}}\alpha_{1}\gamma^{\beta_{1}}x^{\delta \beta_{1}}=L^{\delta \beta_{1}}\alpha_{1}\gamma^{\beta_{1}}$$

Note that the distribution of xylem volume is constant with axial position. This stems from the pipe model hypothesis.

Axial distribution of xylem volume is

(A27) $$V_{p}(x)=nA_{p}={\left(\frac{L}{x}\right)}^{\delta \beta_{1}}\alpha_{2}\gamma^{\beta_{2}}x^{\delta \beta_{2}}=L^{\delta \beta_{1}}\alpha_{2}\gamma^{\beta_{2}}x^{\delta (\beta_{2}-\beta_{1})}$$

Axial distribution of nitrogen in the xylem is

(A28) $$\begin{array}{l} V_{x}(x)=nA_{x}\rho_{N,\;x}={\left(\frac{L}{x}\right)}^{\delta \beta_{1}}\alpha_{1}\gamma^{\beta_{1}}x^{\delta \beta_{1}}\alpha_{3}\gamma^{\beta_{3}}x^{\delta \beta_{3}}\\ \;=L^{\delta \beta_{1}}\alpha_{1}\alpha_{3}\gamma^{\beta_{1}+\beta_{3}}x^{\delta \beta_{3}} \end{array}$$

Axial distribution of nitrogen in the phloem is

(A29) $$\begin{array}{l} V_{p}(x)=nA_{p}\rho_{N,\;p}={\left(\frac{L}{x}\right)}^{\delta \beta_{1}}\alpha_{2}\gamma^{\beta_{2}}x^{\delta \beta_{2}}\alpha_{4}\gamma^{\beta_{4}}x^{\delta \beta_{4}}\\ \;=L^{\delta \beta_{1}}\alpha_{2}\alpha_{4}\gamma^{\beta_{2}}\gamma^{\beta_{4}}x^{\delta (\beta_{2}-\beta_{1}\text{+}\beta_{4})} \end{array}$$

Axial distribution of xylem conductance is

(A30) $$\begin{array}{l} V_{x}(x)=nA_{x}\rho_{c,\;x}r_{x}^{2}={\left(\frac{L}{x}\right)}^{\delta \beta_{1}}\alpha_{1}\gamma^{\beta_{1}}x^{\delta \beta_{1}}\alpha_{7}\gamma^{\beta_{7}}x^{\delta \beta_{7}}\alpha_{5}^{2}\gamma^{\text{2}\beta_{5}}x^{\text{2}\delta \beta_{5}}\\ \;=L^{\delta \beta_{1}}\alpha_{1}\alpha_{7}\alpha_{5}^{2}\gamma^{\beta_{1}\text{+}\beta_{7}\text{+2}\beta_{5}}x^{\delta (\beta_{7}\text{+2}\beta_{5})} \end{array}$$

Axial distribution of phloem conductance is

(A31) $$\begin{array}{l} V_{p}(x)=nA_{p}\rho_{c,\;P}r_{p}^{2}={\left(\frac{L}{x}\right)}^{\delta \beta_{1}}\alpha_{2}\gamma^{\beta_{2}}x^{\delta \beta_{2}}\alpha_{8}\gamma^{\beta_{8}}x^{\delta \beta_{8}}\alpha_{6}^{2}\gamma^{2\beta_{6}}x^{2\delta \beta_{6}}\\ \;=L^{\delta \beta_{1}}\alpha_{2}\alpha_{8}\alpha_{6}^{2}\gamma^{\beta_{2}+\beta_{8}+2\beta_{6}}x^{\delta (\beta_{2}-\beta_{1}+\beta_{8}+2\beta_{6})} \end{array}$$
